# Insights into the structure and function of the hippocampus: implications for the pathophysiology and treatment of autism spectrum disorder

**DOI:** 10.3389/fpsyt.2024.1364858

**Published:** 2024-04-23

**Authors:** Junzi Long, Hui Li, Ying Liu, Xingxing Liao, Zhiqing Tang, Kaiyue Han, Jiarou Chen, Hao Zhang

**Affiliations:** ^1^ School of Rehabilitation, Capital Medical University, Beijing, China; ^2^ Department of Neurorehabilitation, China Rehabilitation Research Center, Beijing, China; ^3^ Division of Brain Sciences, Changping Laboratory, Beijing, China; ^4^ Cheeloo College of Medicine, Shandong University, Jinan, Shandong, China; ^5^ The Second School of Medicine, Wenzhou Medical University, Wenzhou, Zhejiang, China

**Keywords:** autism spectrum disorder (ASD), hippocampus, learning, memory, cognitive map, anxiety, animal models

## Abstract

The hippocampus is one of the brain areas affected by autism spectrum disorder (ASD). Individuals with ASD typically have impairments in hippocampus-dependent learning, memory, language ability, emotional regulation, and cognitive map creation. However, the pathological changes in the hippocampus that result in these cognitive deficits in ASD are not yet fully understood. In the present review, we will first summarize the hippocampal involvement in individuals with ASD. We will then provide an overview of hippocampal structural and functional abnormalities in genetic, environment-induced, and idiopathic animal models of ASD. Finally, we will discuss some pharmacological and non-pharmacological interventions that show positive impacts on the structure and function of the hippocampus in animal models of ASD. A further comprehension of hippocampal aberrations in ASD might elucidate their influence on the manifestation of this developmental disorder and provide clues for forthcoming diagnostic and therapeutic innovation.

## 1 Introduction

Autism spectrum disorder (ASD) is a developmental disability resulting from a combination of genetic and environmental factors, with an estimated prevalence of 0.72% globally (1). The most characteristic symptoms of ASD are impaired social communication and stereotyped or repetitive behaviors (2). In addition, ASD is associated with a typical profile of difficulties across various domains of cognition, including memory, learning, language ability, emotion, and cognitive map creation (3, 4). It has long been recognized that impaired memory for faces, working, and social scenes in individuals with ASD contributes to clinical manifestations of the disorder, such as executive dysfunction (5, 6). The co-occurrence of ASD and learning disability is common, with previous estimates suggesting that as many as three-quarters of individuals with ASD have impaired learning abilities, including language learning skills (7). Language delay is an important feature of autistic children – word and speech learning difficulties are noticeable early in development and continue throughout the school-aged years (8, 9). Individuals with ASD often experience emotional dysregulation, including symptoms of anxiety, which are associated with a range of negative mental and physical health outcomes (10). Data from several navigation and search tasks suggest that people with ASD were slower at learning spatial distribution regularities, less efficient in exploring an environment, and more likely to revisit the area they have already explored (11–13).

The hippocampus has consistently been a brain structure of interest in the seek for physiopathological mechanisms and rehabilitation treatment of ASD, given its important role in memory (14), learning (15), language ability (16), emotional regulation (17), and cognitive map creation (14). For example, recently researchers found that increased recruitment of the hippocampus compensated for decreased connectivity between medial temporal lobes and the posterior medial network during relational encoding tasks, which supported preserved episodic memory in individuals with ASD (18). Previous observations in ASD adults have also suggested that altered hippocampal functioning contributes to difficulties with structural learning that are likely to underlie more complex cognitive processes, including episodic memory, learning processes, and cognitive map creation (19). The language impairments observed in some individuals with ASD are linked to abnormalities in the hippocampal regions (20). Moreover, recent evidence indicates that altered neuronal projection and chemical metabolites in the amygdala-hippocampus region modulate emotional regulation in ASD (21, 22).

Herein, we will first review structural and functional abnormalities in the hippocampus of individuals with ASD and discuss discrepancies in the results of existing studies. In the next part, we will discuss the known genetic, environment-induced, and idiopathic animal models of ASD, mostly in mice and rats, that exhibit hippocampal synaptic plasticity impairments and hippocampus-dependent behavioral deficits. Then, we will summarize the main findings from pharmacological and non-pharmacological interventions for ASD that have been shown to positively affect the structure and function of the hippocampus. Directing more attention to the involvement of hippocampal pathology in the aetiology of ASD and ASD-related behaviors will provide new insights into pathogenic mechanisms, contributing to innovative molecularly or cellularly targeted therapies.

## 2 Hippocampal involvement in individuals with ASD

**Figure 1:**
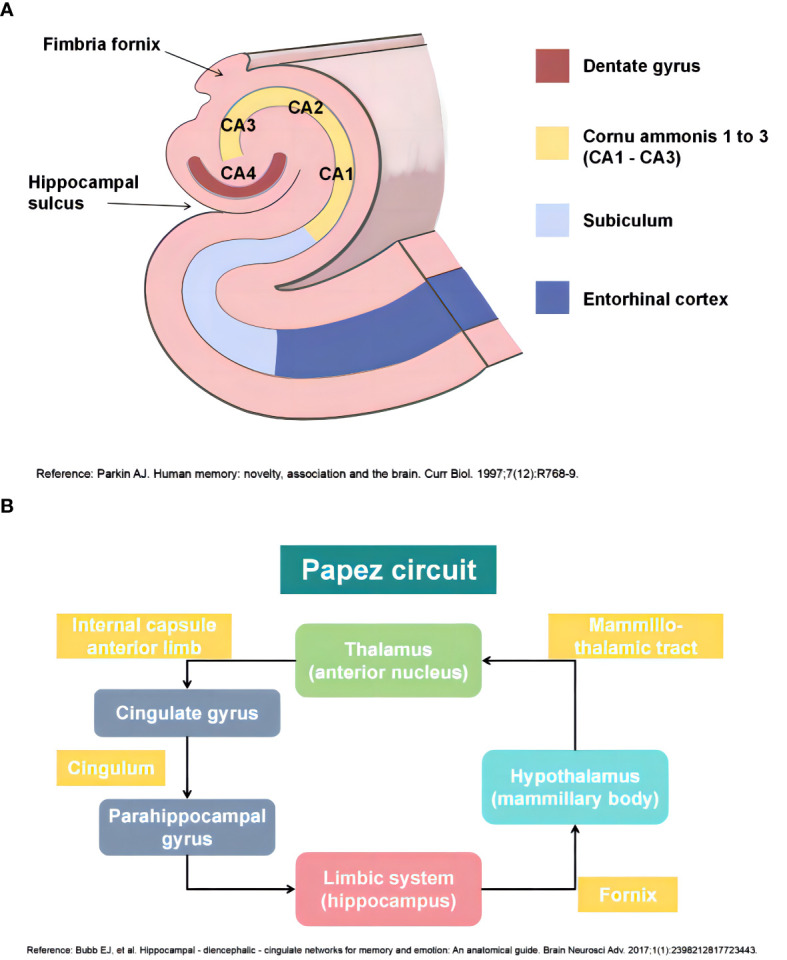
**(A)** Anatomy of the adult human hippocampus (Ref: Parkin AJ. Human memory: novelty, association and the brain. Curr Biol. 1997;7(12):R768-9); **(B)** Papez circuit of the human hippocampus (Ref: Bubb EJ, Kinnavane L, Aggleton JP. Hippocampal - diencephalic - cingulate networks for memory and emotion: An anatomical guide. Brain Neurosci Adv. 2017;1(1):2398212817723443). This figure is created by using Adobe Photoshop and PowerPoint.

The adult human hippocampus, a prominent part of the limbic system, has a three-dimensional tortuous structure that resembles a seahorse because of its arched shape in the axial plane. Based on the cellular composition, the hippocampus can be anatomically divided into four subregions, namely cornu ammonis one (CA1) to cornu ammonis four (CA4) (23). In addition to the hippocampus, the hippocampal formation comprises the dentate gyrus, subiculum, presubiculum, parasubiculum, and entorhinal cortex. The dentate gyrus retains the capacity for neurogenesis throughout an individual’s lifespan, which is widely believed to play a pivotal role in cognitive processes such as learning and memory (**Figure 1A**) (24). The fornix is a primary outflow bundle of the hippocampus and wraps around the thalamus. The hippocampus extends its axons into the fornix to establish synaptic connections with neurons in the mammillary body, which then project fibers via the mammillothalamic tract toward the anterior thalamic nucleus. Subsequently, the cingulate gyrus that contains a large number of myelinated fibers receives a connection from the anterior thalamic nucleus. The cingulate gyrus curves over the corpus callosum and stretches into the parahippocampal region and the entorhinal cortex. In this way, it forms a closed loop by returning to the hippocampal formation. This intrinsic neural pathway, known as the Papez circuit, has long been considered to be responsible for regulating emotions, memories, and learning processes (**Figure 1B**) (25, 26).

The volume of the human hippocampus increases linearly throughout gestation and the first two years after birth, and continues to increase slowly thereafter, suggesting that the crucial developmental period of the human hippocampus is likely to be before the age of two (27, 28). Extensive research has proven that social and behavioral impairments specific to individuals with ASD also emerge between the ages of one-and-a-half and two years, which lead to diagnostic delays until at least three to four years of age (29, 30). It is therefore argued that the developmental timeline of the hippocampus is probably consistent with the behavioral developmental milestones of ASD (31). To sum up, considering the importance of the hippocampus in learning, memory, language ability, emotional regulation, and cognitive map creation, it could conceivably be hypothesized that gaining an understanding of the structural and functional characteristics of the hippocampus in ASD may offer novel perspectives on the pathogenesis and therapeutic targets for ASD.

### 2.1 Hippocampal volume changes

The advancement of neuroimaging methods has significantly increased the ability to observe alterations in the morphology and brain activation patterns of individuals diagnosed with ASD over the past decades. In particular, since the late 1980s, when the first research of ASD using magnetic resonance imaging (MRI) was published, various MRI modalities, including different types of functional MRI, structural MRI, and diffusion tensor imaging, have been reported to effectively facilitate the non-invasive clinical diagnosis of ASD. These publications emphasize that the clinical symptoms of ASD are correlated with volumetric abnormalities in different areas of the brain, including the hippocampus. For instance, extensive MRI research has demonstrated abnormalities in hippocampal volume in individuals with ASD when compared to typically developing people, which is proven to be associated with aberrant autobiographical memory, impaired language skills, social communication deficit, and emotional problems in individuals with ASD (32–34).

Sussman and co-workers (35) have observed a decreased relative volume of the left hippocampus in 72 children and adolescents with ASD. Similarly, a study comparing autistic adults without intellectual disability and healthy community volunteers, found that the reduction in hippocampal volume in the autistic subjects was apparent when corrected for total brain volume (36). Another research based on the ABIDE database also found decreased gray-matter volume in the posterior hippocampus among a large sample of participants with ASD as compared to typically developing controls (32). As these studies all included high-functioning autistic individuals (Full-Scale IQ > 80), the reduction in hippocampal volume is likely to reflect features of autism itself, rather than features of the intellectual deficits that typically accompany autism (36). In other words, autistic traits have a robust correspondence with reduced brain volume in the hippocampus that is related to social processing, working memory, perceptual reasoning, and spatial learning. One possible hypothesis is that high-functioning autistic populations may experience an adaptive reduction in hippocampal volume as a result of their decreased specific functional demands on the hippocampus and altered interactions with the environment (37). Decreased hippocampal volume in ASD individuals was also related to impaired language skills, including verbal learning and memory (32, 38). It has also been suggested that left hippocampal volume is a positive predictor of early language development in females, but not in males (39). Furthermore, it is worth mentioning that in many studies that have reported reductions in hippocampal volume among ASD subjects, only reductions in relative hippocampal volume (percentage of total brain volume) have been observed rather than the absolute volume (35, 36, 40). Numerous studies have now firmly established that individuals with ASD have a significantly larger head circumference and a higher prevalence of macrocephaly than age-matched healthy subjects (41), which could explain the reduced relative hippocampal volume in the ASD group.

Surprisingly, in contrast to the aforementioned findings, an MRI study discovered larger volumes of the hippocampus bilaterally in ASD adults with IQ > 100 when compared to healthy controls (42). Likewise, Xu et al. (43) found evidence of larger absolute hippocampal volume in adults with ASD and adequate intelligence than that of healthy adults based on the ABIDE datasets. The *Intense World Theory* posits that ASD traits may be attributed to the activation of a molecular syndrome, which sensitizes gene expression pathways to excessively respond to environmental stimulation. Under normal circumstances, these pathways facilitate brain development through enriched environments; however, when sensitized, they can lead to accelerated brain development in response to environmental stimuli (44). This theory focuses on the neocortex and the amygdala, but it may also apply to other brain regions, such as the hippocampus. For example, the chronic stress process in ASD enhances amygdala activity, which may initially result in hypertrophy of the hippocampus as it moderates the amygdala activity through multiple reciprocal connections (34). The amygdala and hippocampus also play a major role in olfaction. Research has shown that the severity of taste and smell dysfunction is inversely related to the hippocampal volume, and females with ASD have more severe taste and olfactory impairment than males (45). These theories reflect characteristic behavioral variations in ASD and remain to be elucidated in the future. Another possible hypothesis suggests that the increased size of the hippocampus in people with ASD may be due to an enhancement in experience-dependent function (43, 46). Indeed, individuals with ASD have been reported to excel in certain cognitive domains for which the hippocampus is responsible (e.g. visuospatial abilities) compared to typically developing individuals, but these differences may be driven by task demands (31). Current scientific opinion still holds that ASD can lead to a range of hippocampus-related dysfunctions, which are linked to abnormalities in the volume of the hippocampus (32–34). The assertion that individuals with ASD are more proficient than their neurotypical peers in a specific hippocampus-dependent function is a limited occurrence and cannot be generalized to the entire autistic population (4).

In addition to these observations, a number of researches have reported no difference in the hippocampal volume between ASD individuals and healthy volunteers (47–49). The above inconsistent findings on hippocampal size can actually be attributed to the heterogeneity of ASD individuals. For example, the age range is a significant confounding factor because individuals with ASD appear to consistently have larger hippocampus than their healthy peers from childhood to adolescence (46), but in adulthood, their hippocampus begins to decrease (36), or in some cases, remain stable (47). Although several studies have reported no correlation between age and hippocampal volume in individuals with ASD (35, 42), this may be because of the broad age range of participants, which appears to neutralize the disparities. Second, sex is another important determinant of hippocampal volume in people with ASD. Possibly due to the higher prevalence of ASD in males, existing studies have included a larger sample of male participants. It has been reported that male ASD subjects had faster hippocampal volume growth and greater hippocampal asymmetry than female subjects (50, 51). Relative volumes of both the left and right hippocampus were smaller in females with ASD than in age-matched males (35, 39). Third, many studies have included autism, Asperger’s syndrome, and pervasive developmental disorder (32, 46–48), and a mixed population with varying levels of intellectual disabilities (33, 46, 48) and seizure disorders (52). There are also significant variations in medication statuses, brain MRI scanners, measurement approaches, controls for total brain volume, and anatomical definitions of the hippocampus in related studies. Furthermore, hippocampal abnormalities in ASD could potentially be genetically based, as evidenced by the larger hippocampal volume observed in parents of children with ASD compared to control subjects (53). Due to the high variability in hippocampal volume in typical individuals (54), and the current prevalence of cross-sectional studies with limited sample sizes, considerably more MRI studies will need to be done to determine hippocampal volume changes in ASD, particularly for a specific autistic group with large sample sizes and a longitudinal design.

### 2.2 Hippocampal morphological abnormalities

Postmortem examinations were conducted in ten cases of ASD, comprising eight males and two females, with eight individuals presenting intellectual disability and five individuals exhibiting seizure disorder, ranging from 4 to 29 years old. The findings revealed reduced neural size and increased neuronal density within the hippocampal formation (55–57), similar to typical hippocampal developmental trends. It has previously been observed in an immunohistochemical study, that the density of parvalbumin-, calbindin-, and calretinin-immunoreactive interneurons within the subfields of the hippocampus was increased in cases of ASD compared to controls (58). Increased gray matter density was found in the hippocampal formation and peri-hippocampal cortex of children with ASD, correlating with symptoms of impaired social interaction and mnemonic function in ASD (59, 60). These observations of heightened neuron density in the hippocampal formation could be attributed to incorrect neuronal migration (59). The distorted shape of the dentate granule layer, forming irregular circles and loops, was indicative of abnormal neuronal migration and seemed to be another evidence of the phenomenon described above (61). Additionally, Golgi studies of CA4 and CA1 neurons revealed a noticeable decrease in dendritic branching complexity in two ASD children compared to their age-matched controls. Nevertheless, it remains uncertain whether increased hippocampal neuron density, fewer neuron size volumes, and dendritic branching lead to corresponding reductions in the macroscopic size of the hippocampus. If this is the case, it could account for the decrease in MRI volumes observed in the hippocampus of individuals with ASD. The dentate gyrus development distortion in ASD was manifested by granule cell migration to the molecular layer and the formation of an extra granule cell layer (61). A previous MRI evidence of ASD also suggested that significantly smaller cross-sectional areas of the area dentata than healthy subjects could be attributed to the hypoplasia of the dentate gyrus and CA4 subfield (52). However, caution should be exercised when interpreting these findings as this MRI study and prior neuroanatomical researches have included ASD individuals with seizure disorders that are known to be connected with a decrease in hippocampal volume (62). Moreover, most current autopsy studies have been conducted in males, and it is unclear whether there are sex differences in hippocampal pathological anatomy in ASD. Three-dimensional MRI measurements of hippocampal shape variation in children with ASD indicated an upward curvature in the head and tail of the hippocampus, as well as inward deformation in its medial aspect (48). In terms of morphological structure, the hippocampal regions in children with ASD may be the initial site of alteration and a significant area in brain imaging that can be utilized for diagnosing ASD in children (63). Considering that clinical autistic symptoms were associated with various structural abnormalities in hippocampal regions, additional neuropathological and imaging studies will be needed to obtain the objective criteria for early diagnosis of children with ASD based on the morphological changes of the hippocampus.

### 2.3 Hippocampal blood flow abnormalities

As documented through quantitative MRI and 3D pseudo-continuous arterial spin labeling, cerebral hypoperfusion has been observed in the hippocampus among children with ASD in comparison to healthy controls, which may not be related to sex differences (63–65). Proper brain oxygenation is essential not only for the early development of neurons but also for optimal functioning. Previous research has indicated a positive correlation between hippocampal blood flow and spatial memory performance (66). Therefore, measuring hippocampal blood flow may be an effective method for brain imaging diagnosis in children with ASD. The reduction in cerebral blood flow in the hippocampus also led to a decrease in hemoglobin iron and non-hemoglobin iron in the blood, which ultimately resulted in a decrease in iron content in the hippocampus (63, 65). The hippocampal growth is closely related to blood perfusion, but current findings suggest that reduced blood perfusion and iron ion levels within the hippocampal region do not have a significant impact on the hippocampal volume of ASD children (63–65). One contradictory result was that as compared to healthy controls, high-functioning ASD adults showed a significant cerebral blood flow increase in the right parahippocampal cortex (67). Differences in functional ability and sedative use among the included ASD participants may explain this inconsistency.

### 2.4 Hippocampal metabolite abnormalities

Previous magnetic resonance spectroscopy studies have suggested altered chemical metabolism in hippocampal regions in ASD. Most studies of the hippocampus have reported a trend toward reduced N-acetylaspartate concentrations and N-acetylaspartate/creatine ratios in children with ASD (21, 68, 69). N-acetyl-aspartate synthesis occurs exclusively in the mitochondria of neuronal cells and is widely accepted as a marker for axonal and neuronal integrity and viability (70), so a diminished level of N-acetyl-aspartate may signify an irregularity in hippocampal regional neuronal development and mitochondrial function. This hypothesis was corroborated by a previous study that demonstrated a positive correlation between N-acetyl-aspartate levels in the right hippocampal region and performance IQ in the ASD group (69). However, among adult subjects, Page et al. (71) and O’Brien et al. (72) discovered no variation in NAA levels between the ASD group and the matched comparison group. The impact of age on alterations to metabolites in distinct areas of the brain has been evidenced; for instance, NAA levels are diminished in a neonate and subsequently surge during cerebral development (70). When comparing only boys with and without ASD, there were also no differences in NAA, choline, and creatine concentrations in the hippocampal region (73). People with ASD had a significantly higher concentration of glutamate+glutamine, aka Glx in the amygdala-hippocampal complex than comparison subjects (71), which is consistent with prior data in the auditory cortex of people with ASD (74). As glutamate is the most abundant excitatory neurotransmitter, the pathogenesis of ASD is likely to be associated with increased hippocampal excitability. Previous researches have identified an association between choline/creatine ratio elevation within the hippocampus and the severity of autistic symptoms, including language impairment (69, 75). Similarly, high concentrations of creatine and phosphocreatine in the hippocampal formation of ASD individuals had a significant positive correlation with their aggressive behaviors (70, 76). In sum, it can be hypothesized that disturbances of metabolic substances in the hippocampus are associated with abnormalities in hippocampus-dependent functions.

### 2.5 Hippocampal network connectivity dysfunction

In the study of the autistic human hippocampus, functional MRI has emerged as a powerful tool for mapping disrupted functional connectivity, which denotes the measurement of the temporal synchronization of activity between the hippocampus and other brain regions while a person is at rest or involved in a given cognitive activity. For example, in ASD children and adolescents, the resting-state functional connectivity strength between the left posterior hippocampus and the posterior cingulate cortex has a significant negative correlation with successful memory performance (77). Aberrant circuitry between the hippocampus and posterior cingulate cortex was found to be a common feature in ASD associated with reduced general and facial memory (78). A preliminary neuropsychological and neuroanatomical investigation has indicated that hippocampal and parahippocampal abnormalities are associated with memory deficits in low-functioning ASD individuals, which in turn undermines their learning and language abilities (20). Earlier data from experimental tasks suggested that adults with ASD had particular difficulties with structural learning due to changes in the function of the hippocampus, which played a critical role in the aetiology of ASD (19). During learning, strong functional connectivity was observed between the hippocampus and caudate in both ASD and typically developing adolescents, but this connectivity was positively associated with task performance in ASD and negatively associated with performance in typically developing adolescents (79). Infants with low familial risk for developing ASD exhibited stronger resting-state functional connectivity between the right posterior superior temporal gyrus and the right hippocampus, as well as parahippocampal gyrus, in comparison to high-risk infants. The atypical and immature functional connectivity in high-risk infants could cascade into their later language deficits (80). During the emotional resonance condition, boys with ASD displayed reduced responses in the bilateral hippocampus, indicating potential difficulties with emotional information integration (81). Neural abnormalities within the hippocampus area may potentially place young individuals with ASD at risk for anxiety or other emotional and behavioral dysfunctions (82). Finally, in spatial memory tasks involving the dorsolateral prefrontal-hippocampal circuit, people with ASD showed worse performance than typical controls (83). When performing the virtual reality shopping task, participants with ASD also displayed lower activation in the parahippocampal gyrus, which is implicated in scene recognition and spatial navigation (84). These studies investigate a range of functional domains linked to the hippocampus, indicating that altered functional connectivity between the hippocampal region and other brain networks may account for diverse autism symptoms.

**Figure 2:**
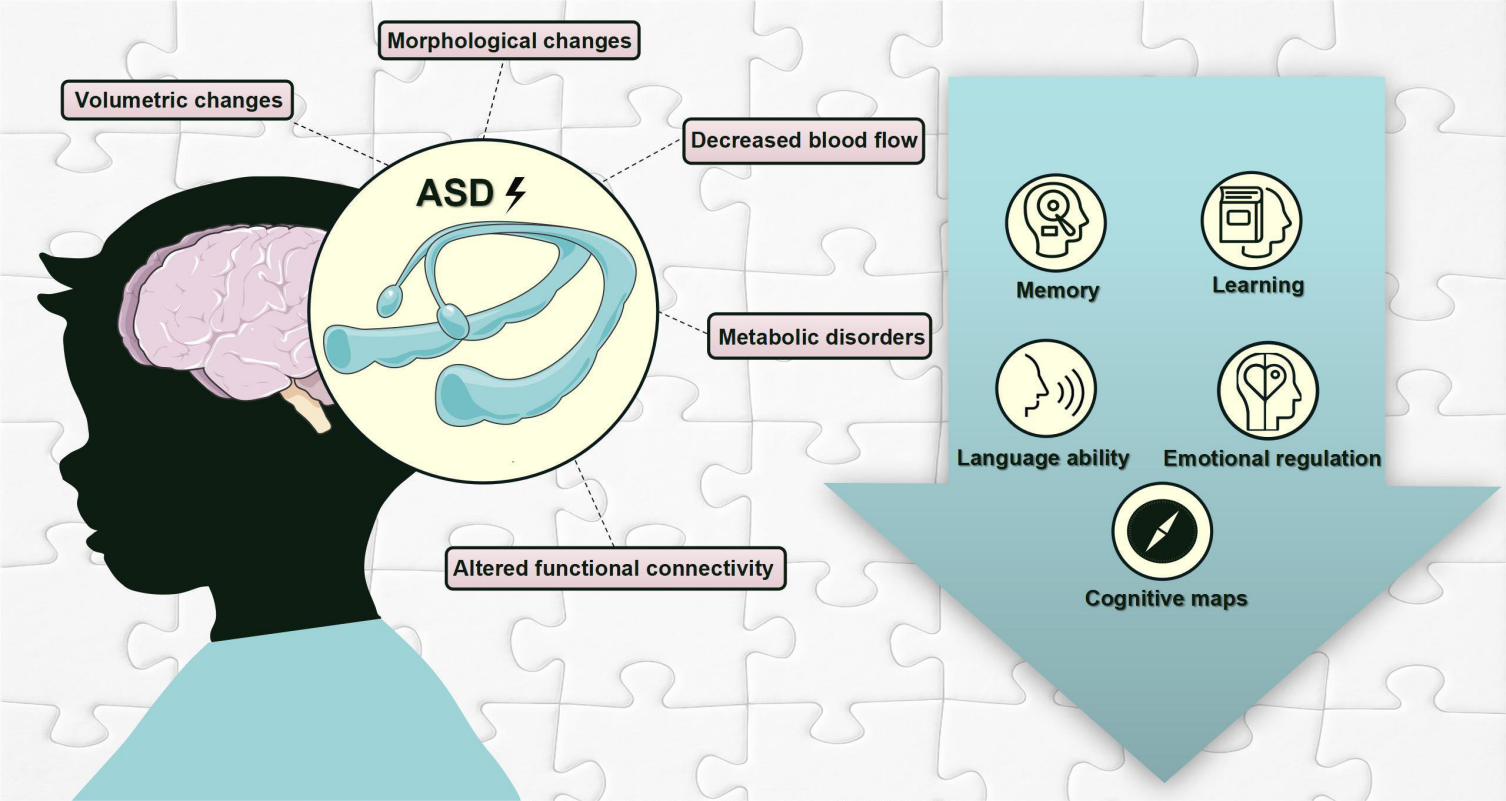
Hippocampal pathological changes and hippocampus-dependent behavioral deficits in individuals with ASD. This figure is created by using PowerPoint.

As discussed above, changes in hippocampal volume, morphology, blood flow, metabolites, and functional connectivity may underlie altered hippocampal functions, including the formation and consolidation of memory, learning, language abilities, emotional regulation, and cognitive map creation (**Figure 2**).

## 3 Abnormalities in the structure and function of the hippocampus in ASD: evidence from animal models

Over recent decades, numerous animal models have been constructed to examine the mechanisms underlying the pathophysiology of ASD, with growing evidence substantiating abnormalities in the structure and function of the hippocampus in ASD. These animal models can be generally categorized into genetic, environment-induced, and idiopathic models.

### 3.1 Animal models based on ASD-associated genes

It is widely accepted that ASD is a heritable disorder with origins in copy number variants, unusual mutations of a single gene, and cumulative effects of particular gene variants (85). To date, hundreds of genetically modified models (especially monogenic models) have intentionally replicated identified human autistic syndromes, both syndromic and non-syndromic ASD. The genetic cause has been clearly defined in cases of syndromic ASD, which often presents alongside ASD-related behavioral phenotypes, and on the contrary, non-syndromic ASD lacks a distinct phenotype (86).

#### 3.1.1 Animal models of syndromic ASD

##### 3.1.1.1 Fragile X syndrome

Transcriptional silencing of the Fragile X messenger ribonucleoprotein 1 (*Fmr1*) gene encoding the Fragile X Messenger Ribonucleoprotein causes fragile X syndrome, the most common monogenic cause of ASD characterized by intellectual and language disabilities (87). Compared to wild-type mice, the dendritic spines of hippocampal neurons in *Fmr1* knockout (KO) mice are longer and thinner, and the density of stubby and mushroom-shaped (mature) spines is lower (88–91). Lazarov and colleagues reported a notable reduction in the number of neural progenitor/stem cells and the subsequent survival of these cells in the subgranular layer of the dentate gyrus in *Fmr1*-KO mice (92). Moreover, hippocampal neurons in *Fmr1*-KO mice had fewer functional synaptic connections, which develop at a slower rate and produce smaller excitatory synaptic currents relative to wild-type controls (93). In agreement, Klemmer et al. (94) discovered that 2-week-old *Fmr1*-KO mice exhibited an early-stage presynaptic phenotype marked by diminutive synaptic structures, decreased number of vesicles per cluster surface, and an overall reduction in the number of synaptic vesicles. *Fmr1*-KO mice also showed a prominent reduction in synaptic density and thickness of postsynaptic density, and an increase in synaptic cleft width (91). On the other hand, while baseline neurotransmitter release and short-term synaptic plasticity are unaffected at CA1 synapse in both younger and older *Fmr1*-KO mice (95), synaptic release probability is excessively elevated in CA1-CA3 areas during repetitive activity, resulting in abnormal short-term plasticity (96). Regarding alterations in long-term plasticity, *Fmr1*-KO mice have specific impairments of glutamatergic signaling in the hippocampus. Abnormally enhanced metabotropic glutamate receptor-dependent long-term depression (mGluR-LTD) in the CA1 region and defective long-term potentiation (LTP) of N-methyl-D-aspartate receptors (NMDA) in the dentate gyrus have been considered as established phenotypes of *Fmr1*-KO mice (95, 97).

These abnormalities observed in the hippocampus may underlie, at least in part, behavioral and cognitive changes of fragile X syndrome animal models. Extensive research has shown that fragile X syndrome murine models have significant deficits in hippocampus-dependent forms of spatial learning and memory as tested using the Morris water maze (91, 98–102). During the novel object recognition test, it was observed that *Fmr1* mutant mice spent significantly more time sniffing the old object and less time exploring the novel object than wild types, suggesting visual recognition memory deficits in these mice (100, 101). Furthermore, *Fmr1*-KO mice had deficiencies in hippocampus-dependent fear memory, characterized by low levels of freezing behavior response to fear conditioning (91, 102). It has previously been noted that *Fmr1*-KO mice had circadian defects involved in hippocampus-dependent memory, which may lead to sleep disturbance (103). The abnormal hippocampal neural activity of *Fmr1*-KO mice during sleep likely leads to adverse consequences for memory processes (104). As mentioned before, neural abnormalities within the hippocampus also cause emotional problems, including anxiety-like behavior. Current experiment results from the open field test and elevated plus maze indicate that *Fmr1*-KO mice exhibit high anxiety levels (91, 105), and mice of the fragile X premutation (CGG repeat sequences between 55 and 200) demonstrate an indication of “social anxiety” (106).

##### 3.1.1.2 Rett syndrome

Rett syndrome, one of the rare ASDs affecting mainly females, is caused by loss-of-function mutations of the X chromosome-linked gene encoding the Methyl CpG binding protein 2 (MECP2) (107, 108). Rett syndrome animal models reproduce typical neurological features of this disorder, including impairments in hippocampus-dependent memory and learning. For example, *Mecp2* mutant mice present deficits in spatial memory and spatial learning in the Morris water maze task (107, 108), the Barnes maze test (109), and the object location test (110). In addition, compared to wild-type mice, *Mecp2* mutant mice showed declines in contextual fear memory when subjected to the fear conditioning task. This was evidenced by their notable decreases in freezing duration, primarily observed at long but not short time scales (107, 108, 111, 112). It is worth noting that the hyperactive hippocampal network (i.e. an imbalance of synaptic excitation/inhibition in hippocampal neurons) is responsible for learning and memory impairments in Rett syndrome (111, 113, 114). Pervasive spontaneous glutamate release in the hippocampus has been considered a defining characteristic of *Mecp2* KO mice, which contributes to the hyperexcitability of neurons (115). In the hippocampus of symptomatic *Mecp2* mutant mice, Schaffer-collateral synapses exhibited enhanced neurotransmitter release (108), and potentiated glutamatergic synapses (e.g. high surface levels of GluA1) occluded the LTP (116). Moreover, the frequency and amplitude of spontaneous excitatory postsynaptic currents of hippocampal neurons from *Mecp2* KO mice were found to be significantly decreased, revealing a loss of excitatory synaptic response in the inhibitory neurons of the hippocampus (111, 113, 117, 118). The diminished basal inhibitory rhythmic activity in the hippocampus of *Mecp2*-null mice can in turn give rise to a hyper-excitable state of the hippocampal network (119). In morphology, CA1 pyramidal neurons and dentate gyrus granule neurons exhibited delayed dendritic maturation and low dendritic spine density in *Mecp2* mutant mice compared to wild types (114, 120), which could potentially arise from insufficient BDNF expression in hippocampal neurons (109). Taken together, these findings imply that mutation in *Mecp2* causes various forms of hippocampal synaptic plasticity impairment, which in turn affects learning and memory functions.

##### 3.1.1.3 Angelman syndrome

With a high prevalence of comorbid ASD, Angelman syndrome is caused by the deletion of the maternally inherited ubiquitin protein ligase E3A (*Ube3a*) gene in the 15q11-q13 chromosome region, associated with impaired hippocampus-dependent learning, memory, and emotion (121–124). Several animal studies have collectively reported that learning and memory deficits in Angelman syndrome are due to a marked decrease in hippocampal LTP (121–124). Researchers have demonstrated that hippocampal pyramidal cells of Angelman syndrome mice have elongated axon initial segments (125), reduced activity-dependent calcium dynamics (122), and hyperpolarized resting membrane potentials (125), which can be attributed to increased expression of α1-NaKA in the hippocampus. These alternations in intrinsic properties of hippocampal neurons could have driven the hippocampal pathology, namely LTP impairment, and memory and learning deficits revealed by contextual fear conditioning and Morris water maze (121, 122). In addition, increased inhibitory phosphorylation of αCaMKII (123) and elevated Arc expression (124) in hippocampal slices from Angelman syndrome mice also underlie the hippocampal LTP deficits. As another type of synaptic plasticity involved in learning and memory, mGluR-LTD of excitatory synaptic transmission was enhanced in hippocampal slices of *Ube3a*-deficient mice, possibly due to increased synaptic small conductance calcium-activated potassium channel protein 2 levels in the hippocampus (126). As far as emotional problems are concerned, maternal *Ube3a*-deficient mice are under chronic stress and exhibit anxiety-like behaviors. Within the hippocampus, these mice demonstrate susceptibility to glucocorticoid exposure (127), disrupted glucocorticoid receptor signaling (127–129), and reduced number of parvalbumin-positive inhibitory interneurons (128, 129), resulting in chronic stress, hippocampal hyperactivity and ultimately increased anxiety.

##### 3.1.1.4 Tuberous sclerosis complex

Tuberous sclerosis complex is a rare form of ASD that is often accompanied by epilepsy and cognitive deficits, caused by mutations in either of the Tuberous sclerosis complex 1 or 2 (*Tsc1/2*) gene. These genes act as inhibitors of the mTOR signaling, and their mutations lead to hyperactivity of the pathway (130). Like other syndromic ASDs, tuberous sclerosis complex mice also have impairments in hippocampus-dependent spatial learning (131–133), contextual fear memory (131–133), and spatial working memory (131), associated with hippocampal synaptic excitation/inhibition imbalance induced by up-regulated mTORC1 signaling (131, 134). This hippocampal hyperactivity likely results from a reduced synaptic inhibition of pyramidal cells, while the excitatory transmission is unaffected (131, 134, 135). Previous studies have identified a potential link between hippocampal hyperexcitability and epilepsy phenotypes in mice deleting *Tsc1* or *Tsc2* (134, 136), but Koene et al. found the hippocampal excitation/inhibition imbalance only present in the epileptic state, which suggests that these changes in the hippocampus are unlikely to drive epileptogenesis (135). Additionally, dysfunctional glutamate homeostasis (137), impaired astrocytic gap junction coupling (138), and altered potassium clearance (138), as well as microgliosis (139) and astrogliosis (140) in the hippocampus of tuberous sclerosis complex mice, are correlated with seizure onset. Prior studies have noted that mGluR-LTD was not enhanced but rather reduced in tuberous sclerosis complex mutant mice (141–143), indicating divergent synaptic plasticity phenotypes from fragile X syndrome and Angelman syndrome. A possible explanation for this is that the deletion of *Tsc1*/*Tsc2* genes heightened the expression of mGluR5 and exaggerated ERK signaling, which developed a novel mTOR-independent LTP in the CA1 hippocampus (141, 142). Several studies have also reported the enlargement of hippocampal neurons and dendritic spines in mutant mice (143, 144), whilst significantly increased basal cerebral blood flow and oxygen consumption in the hippocampus of rats with tuberous sclerosis complex could partially explain this observation (145, 146).

##### 3.1.1.5 Phelan-McDermid syndrome

As major synaptic scaffolding proteins, SH3 and multiple ankyrin repeat domains protein 1/2/3 (SHANK1/2/3) are highly concentrated in the postsynaptic density of hippocampal excitatory synapses (147–149). Shank family genes (*Shank1/2/3*), especially the Shank 3, are well-known ASD-related genes. Deletions or mutations of the *Shank3* gene can lead to Phelan-McDermid syndrome, characterized by autistic behavior, intellectual disability, and speech delay (150). It has been reported that the disruption of major *Shank3* isoforms in mouse/rat models decreases levels of other post-synaptic density scaffolding components and glutamatergic receptors in the hippocampus, including the HOMER1 (147–149), PSD95 (148), mGluR5 (148), GluA1 (149), and GKAP (149). At the level of hippocampal synaptic morphology, these animals had smaller postsynaptic density structures (151), lower spine density (149, 151, 152), and longer dendritic spines (149, 151) as compared to wild types. Unlike these profound synaptic changes, however, there have been no distinct alterations in the frequency and amplitude of miniature excitatory (149, 153–155) and inhibitory (149, 154, 155) postsynaptic currents, as well as field excitatory postsynaptic potentials (147, 156) in CA1 pyramidal neurons from mice and rats lacking different *Shank3* isoforms. These data indicate that basal synaptic transmission, neurotransmitter release probability, and short-term plasticity at hippocampal synapses may be preserved in Phelan-McDermid syndrome. Indeed, *Shank3* is critical for long-term hippocampal synaptic plasticity. *Shank3*-deficient mice and rats have reduced LTP (147, 149, 155–157) but unaltered LTD (155–157), and a hippocampal excitation/inhibition imbalance (153, 154). These deficits result in impaired social recognition memory (147, 156, 158), object location memory (147, 149, 155), and spatial learning and memory (149, 152, 153, 155, 156), particularly affecting long-term memory processes. In contrast to the above observations, Peça et al. (159) have reported that the frequency and amplitude of miniature excitatory postsynaptic currents and Morris water maze performance in *Shank3*B mutant mice are comparable to those of controls. Similarly, Cope et al. (160) have found impaired social but not object location memory in *Shank3*B KO mice. Contradictory findings across these studies may be attributed to mutations of various *Shank3* isoforms. This hypothesis is backed by recent transcriptomics investigating gene dosage-differential changes in the hippocampus of *Shank3* mutant mice (161).

#### 3.1.2 Animal models of non-syndromic ASD

##### 3.1.2.1 Neuroligin (Nlgn)

The NLGN family of synaptic cell adhesion molecules is fundamental in regulating excitatory and inhibitory synapses. There are four isoforms (*Nlgn1-4*) expressed in rodents or humans, all of which are linked with ASD symptoms. *Nlgn1* is predominantly localized to glutamatergic synapses, and *Nlgn1* overexpression in mice enhances the number and maturity of excitatory synapses and spines in the CA1 region (162). As for *Nlgn1*-KO mice, it is highly probable that their hippocampal NMDAR-LTP deficits (163, 164) result from diminished glutamate receptor functions, including reductions in NMDAR-mediated excitatory transmission (163, 164), expression levels of synaptic AMPA and NMDA (163), and the NMDAR/AMPAR ratio (164, 165) in perforant path-granule cell synapses and CA1 pyramidal neurons. In contrast, the *Nlgn2* is principally localized to inhibitory GABAergic synapses with a key role in enhancing inhibitory but not excitatory synaptic function (165). *Nlgn2*-deficient mice displayed reduced postsynaptic gephyrin and GABAAR cluster numbers in the dentate gyrus, decreased inhibitory GABAergic synaptic transmission, and increased granule cell excitability (166). This observation matches those observed in recent studies that mice overexpressing *Nlgn2* have a reduced hippocampal excitation/inhibition ratio, thereby inhibiting their aggressive behaviors and impairing spatial memory performances (167, 168). *Nlgn3* is the only NLGN isoform that is found in both excitatory and inhibitory synapses. In *Nlgn3* KO mice, the number of excitatory synapses in the CA1 stratum oriens (169) and neuronal excitability in the CA2 area (170) were increased. Deleting *Nlgn3* also reduced hippocampal gamma oscillations and sharp wave ripples, which could lead to abnormal fear memory retention and extinction (170, 171). *Nlgn3*-R451C mutant mice exhibited large increases in both excitatory (172, 173) and inhibitory (174) synaptic transmission in the hippocampal CA1 region, and particularly the increased NMDA/AMPA ratio may have enhanced NMDAR-dependent LTP (172, 173). This also accords with several observations, which showed that these mice have better spatial learning and memory performance than wild-type controls (173, 174). However, it has also been shown that the *Nlgn3*-R451C mutation can cause loss-of-function effects in neonatal mice, characterized by premature hyperpolarizing effect of GABA at immature hippocampal MF-CA3 synapses and fail to express spike time-dependent LTP (175). The specific subcellular localization of *Nlgn4* in synapses is not fully understood, but *Nlgn4* is certainly expressed in the mouse hippocampus (176). A prior study has shown that the loss of *Nlgn4* caused postsynaptic changes at inhibitory synapses and aberrant inhibitory neurotransmission, heavily disrupting γ-oscillations in the CA3 region of the mouse hippocampus (176). Unexpectedly, Muellerleile and colleagues discovered increased network inhibition within the dentate gyrus of adult *Nlgn4* KO mice but unaltered in neonatal *Nlgn4* KO mice (177). Guneykaya et al. (178) found that hippocampal γ-oscillations were disrupted and hippocampal microglia density was reduced only in male *Nlgn4* KO mice. Hence, contradictory results in the hippocampal inhibitory state may partly be explained by sex-dependent and age-related impacts of *Nlgn4* loss.

##### 3.1.2.2 Phosphatase and tensin homolog detected on chromosome ten (Pten)

Like *Tsc1*/2, the *Pten* gene functions as a mTOR pathway negative regulator, and alteration of this pathway is involved in ASD pathogenesis. Activation of mTOR via *Pten* deletion from hippocampal dentate granule cells re-initiates additive growth, which leads to hypertrophied neurons (179–182), enlarged mossy fiber axons (179, 180, 183), elongated dendrites (179, 180, 184) and increased dendritic spine density (179–181, 184). These hippocampal morphological changes partly underlie the macrocephaly and epilepsy that are notable features of inherited *Pten* mutations. The observed increase in spontaneous excitatory synaptic current frequency (particularly in females) (181, 184) and field excitatory postsynaptic potential slope (185, 186) suggests increased excitatory synapses on cells and enhanced basal synaptic transmission in the hippocampus of *Pten* KO mice. The increased epileptogenic activity of *Pten* KO mice is largely due to hippocampal hyperexcitability (182, 184). More importantly, these mouse models display impaired hippocampal LTP and LTD (185, 187), as well as spatial memory (187) during postnatal development, which has been shown to precede the appearance of their morphological abnormalities (185).

##### 3.1.2.3 Cyclin-dependent kinase-like 5 (Cdkl5)

Mutations in the X-linked *Cdkl5* gene cause severe neurodevelopmental disorders marked by early-life autistic behaviors and intractable epilepsy (188). The *Cdkl5* is highly expressed in the hippocampus, and its deficiency in mice reduces dendritic length, branches, and maturation of hippocampal pyramidal and granule neurons (189–192). Meanwhile, *Cdkl5* KO mice exhibit an elevated incidence of newborn cell apoptosis within the hippocampal dentate gyrus leading to diminished granule neuron counts (189, 190), coupled with accelerated senescence and death of hippocampal neurons during the aging process (191). Strikingly, the increase in apoptosis is paralleled by the rapid proliferation of neuronal precursor cells in the dentate gyrus, which modulates the equilibrium between precursor proliferation and survival (190). Additionally, it is noted that hippocampal neurons of *Cdkl5* KO mice demonstrate heightened susceptibility to neurotoxicity, excitotoxicity, and oxidative stress (192, 193), implying that the absence of *Cdkl5* augments neuronal vulnerability. These neuroanatomical alterations are associated with hippocampus-dependent learning and memory impairment observed in multiple tasks (189–191). The robust seizures in *Cdkl5* KO mice have been demonstrated to be correlated with microglial activation (192), BDNF-TrkB signaling enhancement (194), and postsynaptic overaccumulation of GluN2B-containing NMDAR (195) in the hippocampus.

### 3.2 Animal models of environment-induced ASD

It is clear now ASD etiology involves both genetic and environmental factors or their possible combinations. Exposure of animals to given chemicals, toxins, viruses, and other agents during gestation can induce models of ASD in their offspring.

#### 3.2.1 Valproic acid induced animal models

Valproic acid (VPA) is a commonly used anti-epileptic or mood-stabilizing drug but is classified as a human teratogen. In animal studies, typical ASD models in newborn mice or rats have been simulated by exposing their mothers to VPA during pregnancy (196). Similar to the ASD traits in humans, male animals might be more susceptible to VPA-induced ASD than females. For instance, male VPA mice exhibited higher locomotor activity and lower social ability index than females, potentially attributed to increased hippocampal cell atrophy and heightened expression of the 5-HT2A receptor protein in the hippocampus (197). The VPA exposure in mice or rats can induce several autism-like behaviors related to hippocampal functions, including impairments in spatial learning and memory (198–206), visual recognition memory (198, 203, 207–209), working memory (208, 210), and emotional regulation (199, 200, 204–206, 211–213). It is widely documented that exposure to VPA notably increases levels of the pro-inflammatory markers (IL-1β, TNF-α, IL-6, IFN-γ, IL-17, TGF-β) and reduces levels of the anti-inflammatory marker (IL-10) in the hippocampus (199, 208, 211, 213–216). The hippocampal neuroinflammatory state is primarily observed in young ages, perhaps resulting from microglia and astrocyte activation that started in the early postnatal developmental stages. In adult mature VPA rats/mice, however, changes in the expression of neuroglial markers in the hippocampus seem to be mild, with an amelioration of the neuroinflammatory phenotype (212, 217, 218). After exposure to prenatal VPA, biochemical markers associated with neuronal oxidative/nitrosative stress such as MDA, TBARS, and NO were found to be significantly increased contrary to markers such as GSH, SOD, and CAT in the hippocampal regions (198, 202, 212–215, 219). At the same time, increased oxidative stress is accompanied by aberrant mitochondrial electron transport chain enzyme activity, reduced ATP levels, and ultrastructurally destructed mitochondria in the hippocampus (198, 212). Exposure of mice or rats to VPA activates mTOR and Notch signaling, which amplifies autophagic deficiency in the hippocampus, characterized by decreased expression levels of Beclin1 and LC3-II and a small number of autophagosomes (203, 210). Previous studies evaluating hippocampal excitatory/inhibitory imbalance found that VPA exposure enhanced excitatory glutamatergic and impaired inhibitory GABAergic synaptic transmission, with a decrease in the GABA/glutamate ratio (201, 206). Compared to controls, VPA-induced rats showed a significantly larger number of hippocampal apoptotic neurons, accompanied by increased levels of the apoptotic markers “Bax, caspase-3 and p53” and decreased levels of the antiapoptotic marker “BCL2” (201, 204, 219–221). Moreover, reductions in the hippocampal levels of BDNF, synapsin-IIa, DCX, and pCREB are strongly implicated in ASD as these proteins play significant roles in neuronal formation, synaptic transmission, neuroplasticity, and neurogenesis (201, 213–215, 221). Taken together, these pathophysiological processes significantly weaken hippocampal neuron viability in VPA-exposed mice and rats. Furthermore, VPA exposure significantly altered the expression of multiple ASD candidate genes in the hippocampus: *Shank3* (212), *Shank2* (209), *Nlgn3* (212), and *Pten* (222). This observation further supports that high-risk ASD genes can also be altered by environmental factors – gene-environment interactions have a pivotal role in the pathophysiology of ASD.

#### 3.2.2 Maternal immune activation induced animal models

Maternal immune activation (MIA) during pregnancy increases the risk of the unborn fetus developing ASD later in life (223). The most commonly used methods for emulating MIA models of ASD involve the intraperitoneal administration of lipopolysaccharide (LPS) and polyinosinic: polycytidylic acid (poly (I: C)) during gestation. The administration of LPS can induce sex-dependent alterations in hippocampal volume, neuronal morphology, and gliovascular maturation. Compared with female LPS-induced mice, the male LPS group showed a larger size of hippocampus (224), higher hippocampal neuronal spine density (224, 225), and lower vascular coverage of astrocytic end-feet (226) associated with their reduced interest in social novelty (224, 225). Microglial activation and astrogliosis could be functionally important in altering hippocampus-dependent learning and memory performance observed in LPS-induced rat models (227, 228). Surprisingly, at the early postnatal stage, exposure to LPS had no negative effects on hippocampal cellular or tissue morphology but instead stimulated nerve growth by promoting cell proliferation (228), increasing the number of spines (225), and raising the density of mossy fiber synapses (229) in the hippocampal area. It seems possible that these results are due to the M2-biased microglia polarization at the acute inflammatory phase, which releases excessive anti-inflammatory cytokines and growth factors (228). In contrast, MIA induced by poly (I: C) did not alter the density of Iba1+ microglia (or GFAP+ astrocytes), nor did it modify their activation phenotypes in the hippocampal formation of the offspring (230, 231). However, prenatal exposure to poly (I: C) increased hippocampal IL-6 and IL-1β levels, resulting in the promotion of hippocampal kindling epileptogenesis (230, 232). Additionally, the offspring of poly (I: C)-exposed mice displayed a substantial reduction in the relative density of hippocampal pre- and postsynaptic proteins (synaptophysin, bassoon, PSD95, and SynGap) and changed the firing traits of hippocampal place cells in adult offspring (230, 233). The hippocampal synaptic deficits could alter the electrophysiological properties of hippocampal cells, hence affecting the firing activity of hippocampal neurons. These changes may underlie the spatial memory impairments found in MIA mice following poly (I: C) injection (233, 234).

#### 3.2.3 Air pollution induced animal models

Gestational exposure to air pollution can increase the incidence of ASD in offspring (223). Hippocampal transcriptome data revealed that gestational nanosized particulate matter (PM) exposure induced multiple differentially expressed genes in young adult offspring. The stratification by sex revealed a twofold increase in the number of differentially expressed genes in males compared to females, and there was male-specific enrichment of differentially expressed genes involved in serotonin receptor signaling, cAMP-mediated signaling, and endocytosis (235). Other studies have also found that PM2.5-induced mice have aggravated hippocampal neuroinflammation, with elevation in NF-κB, TNF-α, and IL-1β levels (236) and microglial activation (237). Meanwhile, these mice exhibited impaired spatial learning and memory, associated with disrupted hippocampal synaptic ultrastructure, decreased hippocampal neurogenesis, and increased hippocampal neuronal apoptosis (236). These findings can explain the reduction in hippocampal size and structural integrity after exposure to airborne PM (237, 238).

### 3.3 Animal models of idiopathic ASD

As genetic and environment-induced models cannot accurately replicate all the pathological features of ASD, strains of mice and rats have been developed using idiopathic models, which display robust and well-replicated behavioral characteristics of ASD such as social deficits and repetitive behaviors. The BTBR T+Itpr3tf/J (BTBR) strain is one of the most valid models of idiopathic ASD, and inbred strain C57BL/6J is often used as a control for BTBR (239).

#### 3.3.1 BTBR animal models

Recently, the BTBR inbred mouse strain has gained popularity as a rodent model of ASD. In addition to the core symptoms of ASD, BTBR mice also display learning and memory impairments in various settings (240–242). Several histological observations and MRI assessments support separated hippocampal commissure and increased hippocampal volume in BTBR mice relative to controls, and these anatomical changes may underlie their behavioral phenotypes (243–246). Moreover, the hippocampus has lower 5-HT, acetylcholine, dopamine, and histamine content in the BTBR animals than in the C57BL/6J strain (246–248). In particular, immunofluorescent labeling of 5-HT transporter axons revealed a reduction in the density of innervation to the hippocampus in BTBR mice (246). Premature changes in hippocampal neuronal excitability, involving elevated ERK signaling (248) and increased GABAergic neurotransmission (249) during neonatal development in the BTBR mice, may also contribute to the high susceptibility to epilepsy and aggressive behaviors observed in these mice (248, 250). As for adult and aged BTBR animals, significant reductions in mRNA or protein levels of BDNF, as well as neurogenesis in the hippocampus have previously been reported (241, 245, 250).

**Figure 3:**
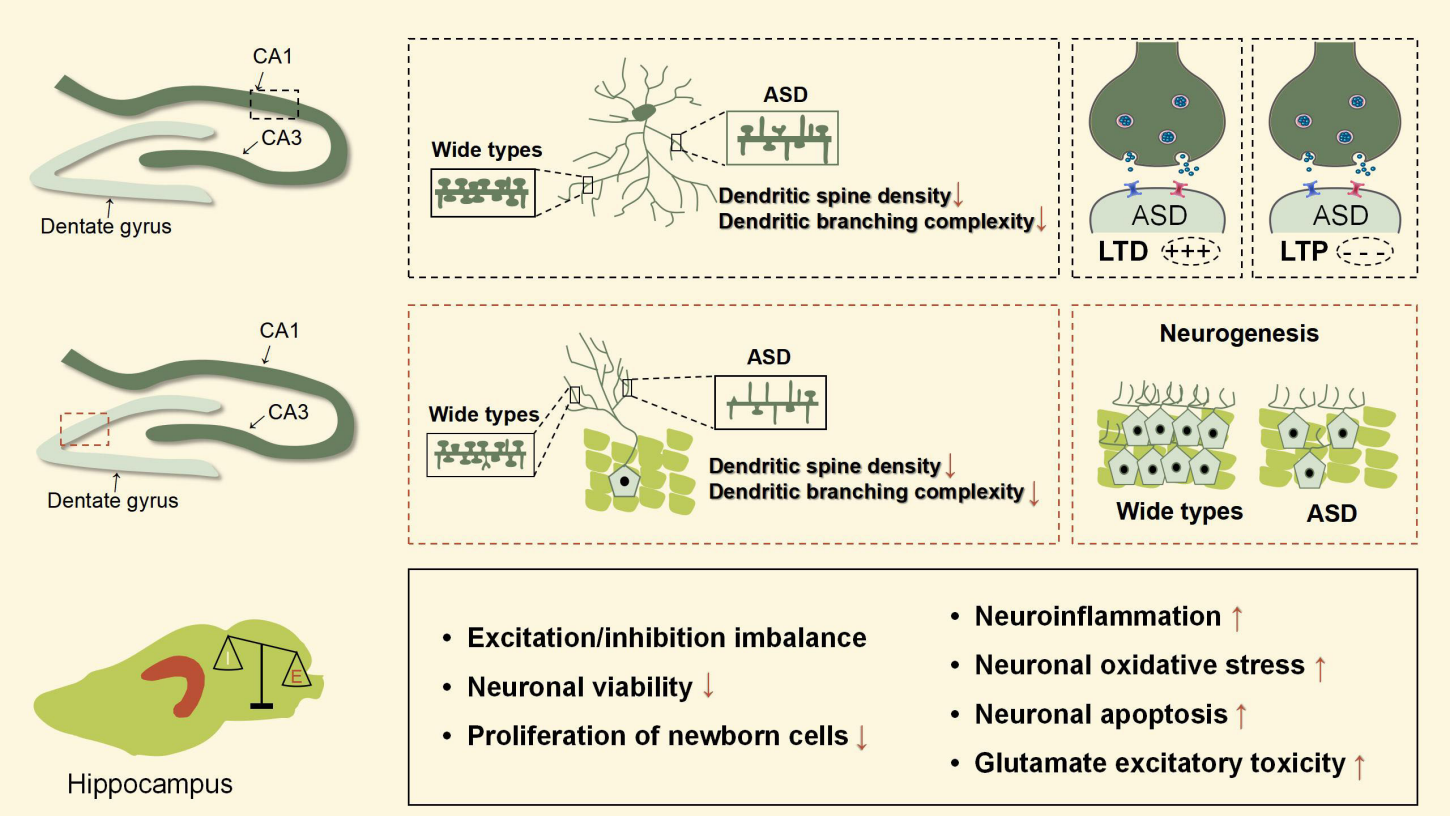
Hippocampal pathophysiological processes of ASD animal models. This figure is created by using PowerPoint.

**Figure 4:**
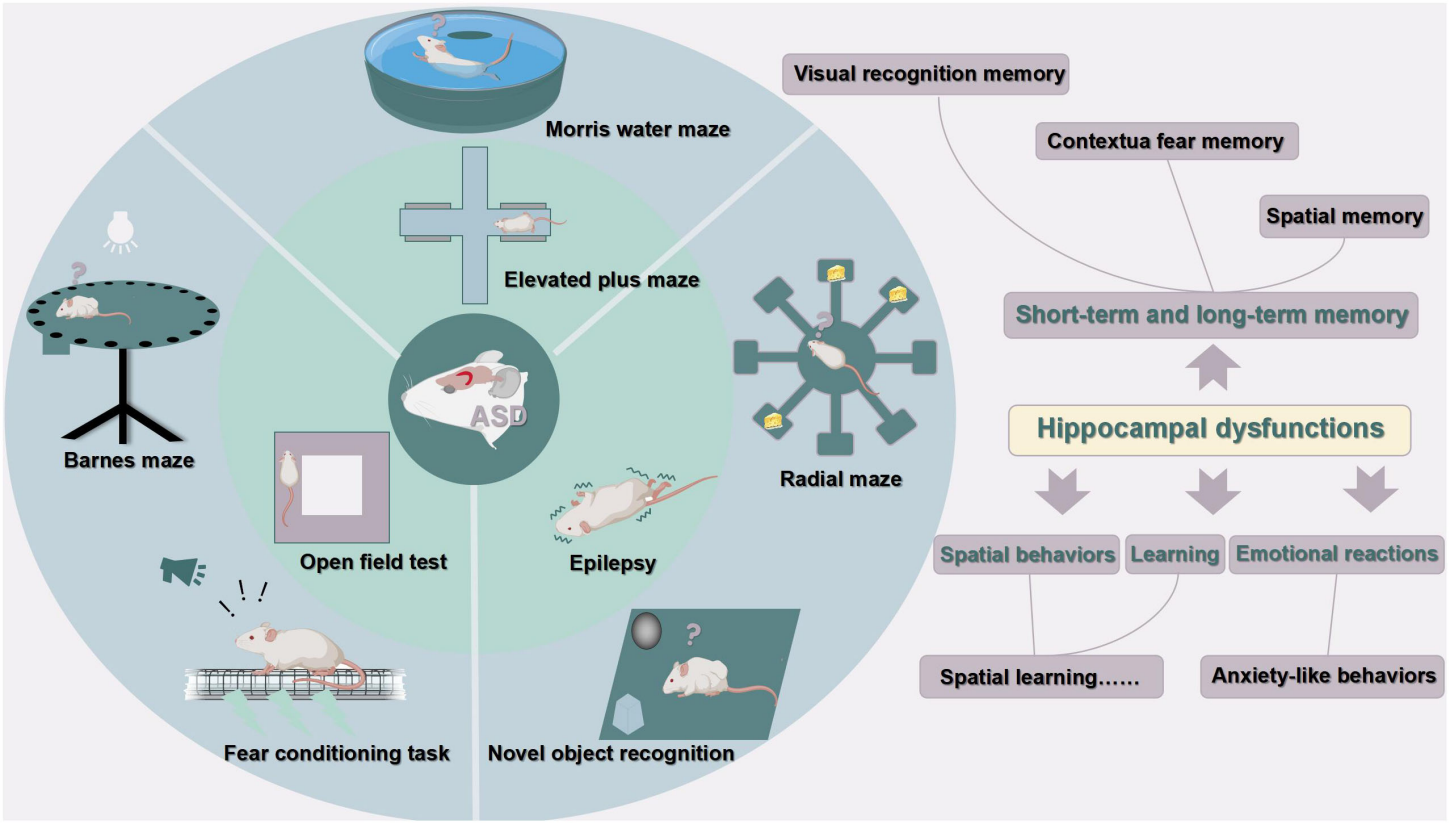
Hippocampal dysfunctions in ASD animal models. This figure is created by using PowerPoint.

To sum up, hippocampal excitatory/inhibitory imbalance is one of the most important pathological mechanisms in ASD. These animal models mainly show impairments in dendrite morphology, neurogenesis, neuronal viability, LTP, and LTD in the hippocampus, leading to dysfunction in learning, memory, emotional regulation, and spatial ability. **Figures 3**, **4** were used to depict some common hippocampal structural and functional impairments in rodent models of ASD, but it is important to note that each model has its own characteristics of hippocampal dysfunctions and cannot be generalized. In a word, the above animal studies suggest that the hippocampus is strongly implicated in the pathophysiology of ASD and should be considered as an important target for future therapeutic approaches.

## 4 Therapies that positively influence the structure and function of the hippocampus in ASD animal models

**Figure 5:**
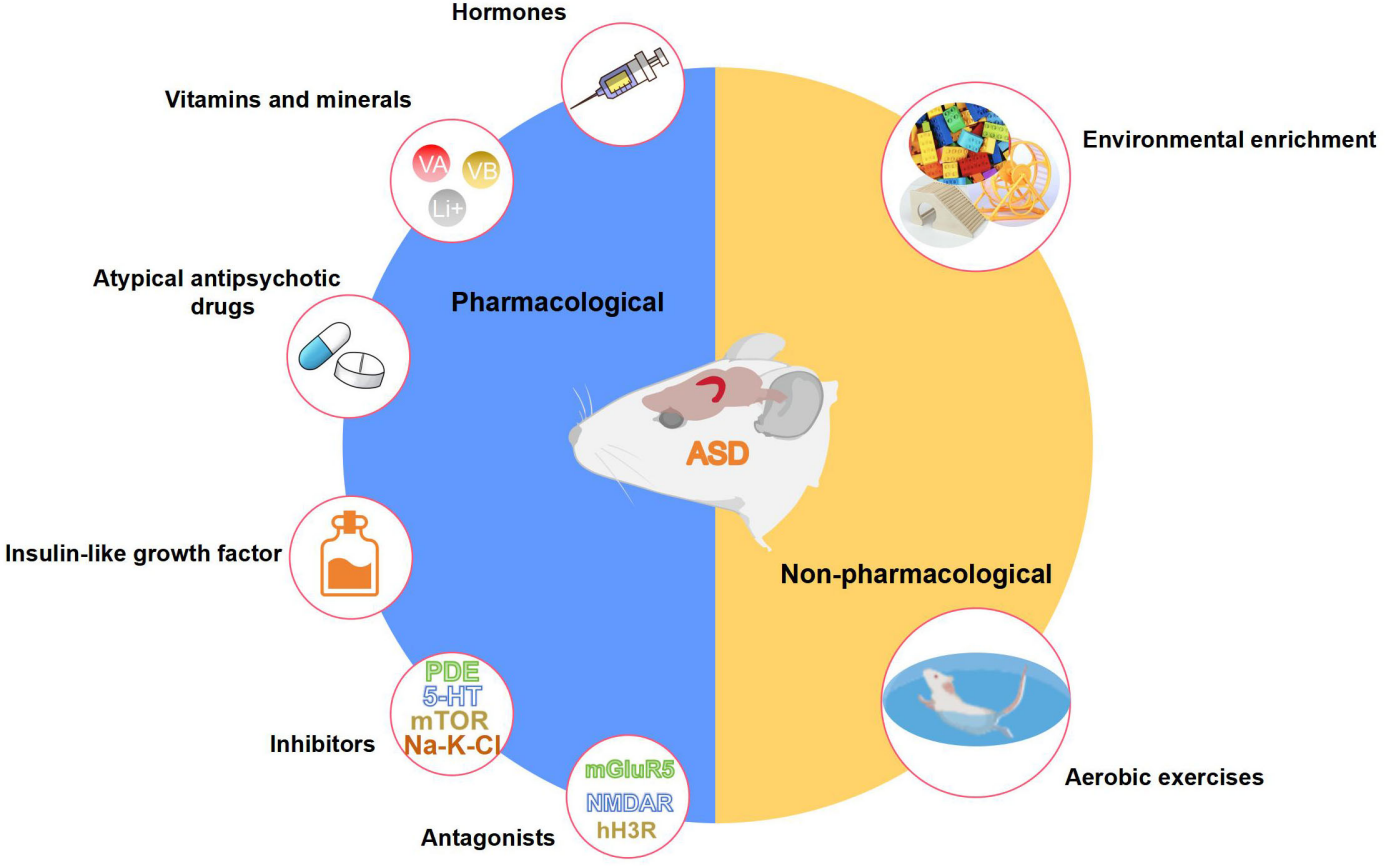
Pharmacological and non-pharmacological interventions that positively affect the structure and function of the hippocampus in ASD animal models. This figure is created by using PowerPoint.

To date, amounts of pharmacological and non-pharmacological interventions that positively impact hippocampus-dependent cognitive functions have been identified through animal experimentation as potential treatments for ASD (**Figure 5**). Pharmacological interventions mainly include hormones, vitamins and minerals, atypical antipsychotic drugs, phosphodiesterase inhibitors, selective serotonin reuptake inhibitors, mTOR inhibitors, mGluR antagonists, NMDAR antagonists, histamine H3 receptor (H3R) antagonists, and insulin-like growth factor. Non-pharmacological interventions mainly include aerobic exercise and environmental enrichment. **Table 1** presents elaborate cellular and molecular modifications in the hippocampus of animal models of ASD following each intervention.

**Table 1:** Main effects of each intervention on cellular and molecular changes in the hippocampus of ASD animal models.

Intervention		Animal model	Cellular and molecular changes in the hippocampus	References
Hormone	Oxytocin	*Shank3* mutant mouse	Protein level: Synapsin I↔, PSD95↔ Gene expression: Synapsin I↑, Psd95↑, Nlgn3↑, Nlgn2↑, Nrxn1β↑, Nrxn2α↑, Nrxn2β↑, Rhob↑, Rac1↑, Pak1↑, Pak2↓	(251)
		VPA-exposed rat	Number of parvalbumin-positive interneurons↑ Enzymatic activities of the mitochondrial electron transport chain↔, 4-HNE protein↔	(198)
			Gene expression (transcriptome analysis): Kcnj13↑, KI↑, Prelp↑, Sostdc1↑, Clic6↑, F5↑, Sclc4a5↑, Mdk↑, Folr1↑, Mmp14↑, Tmem72↑, Kcne2↑, Sfrp1↑, Mt-nd3↓, Slc19a3↓	(252)
	Melatonin	VPA-exposed rat	Protein level: p-CaMKII↑, p-Synapsin I↑, p-NMDAR1↑, p-MARCKS↑, p-PKA↑, p-GluR1↑	(253)
Vitamin and mineral	Vitamin A	VPA-exposed rat	Gene expression (RNA sequencing): lncRNA NONRATT021475.2↓, desert hedgehog gene↓	(254)
	Vitamin B6, folic acid and vitamin B12	PM_2.5_-exposed mouse	Number of damaged mitochondria↓, synaptic cleft↓, PSD thickness↑, synaptic active area length↑, apoptotic ratio↓, neurogenesis↑ Protein level: MDA↓, SOD↑, GSH-Px↑, GSH↑, NF-κB↓, TNF-α↓, IL-1β↓, Caspase-3↓ Gene expression: NF-κB↓, TNF-α↓, IL-1β↓	(236)
	Folic acid	BTBR mouse	Protein level: GFAP↓, Iba-1↓, IL-1β↓, IL-6↓, IL-18↓, TNF-α↓, MDA↓, SOD↑, GSH-Px↑, GSH↑, p-CaMKII↑, p-CREB↑, GPx4↑, Fpn1↑, SOD1↓, TFR↓	(240)
	Selenium	BTBR mouse	Number of viable neurons↑ Protein level: 5-HT↑, Dopamine↓, glutamate↓, IL-6↓, IL-18↓, TNF-α↓ MDA↓, SOD↑, GSH-Px↑, GSH↑, CAT↑ Gene expression: IL-6↓, IL-18↓, TNF-α↓	(241)
	Lithium	*Fmr1* mutant mouse	Protein level: GSK-3β↔, pS202-Tau/Tau↓, Tau↓, pS2448-mTOR/mTOR↓, mTOR↔	(255)
		VPA-exposed rat	Number of Iba-1 positive cells↓, IL-6↓	(211)
Atypical antipsychotic drug	Aripiprazole	VPA-exposed mouse	Dendritic spine density↑	(207)
			Number of Nissl-positive cells↑	(210)
		VPA-exposed rat	Number of intact neurons↑, Number of neurofibrillary tangles↓, Nissl’s granules optical density↑ Protein level: glutamate↓, GABA↑, BDNF↑, Caspase-3↓, Bax↓, Bcl-2↑, GFAP↓, CREB↔, p-CREB↔ Gene expression: Glt-1↑	(201)
	Risperidone	VPA-exposed mouse	Dendritic spine density↑	(207)
		VPA-exposed rat	Number of viable neurons↑ Protein level: cytochrome-c↓, lactate dehydrogenase↓, caspase-3↓, MDA↓, GSH↑, Bcl-2↑, Gene expression: Adar2↑, GluA2 Q:R↓	(219)
	Olanzapine	VPA-exposed rat	Dendritic spine density↑, dendritic length↓, neuron volume↓, number of Nissl-positive neurons↓	(203)
Phosphodiesterase inhibitor	Ibudilast	VPA-exposed rat	Protein level: SOD↑, GSH↑, CAT↑, IL-6↓, IL-1β↓, TNF-α↓, IL-10↑	(199)
	Vinpocetine	VPA-exposed rat	Number of pyknotic and chromatolytic cells↓ Protein level: BDNF↑, synapsin-IIa↑, DCX↑, pCREB↑, CREB↑, pCREB/CREB↑, IL-6↓, TNF-α↓, IL-10↑, GSH↑, TBARS↓	(213)
	Cilostazol	VPA-exposed rat	Protein level: BDNF↑, pCREB↑, IL-6↓, TNF-α↓, IL-10↑, GSH↑, TBARS↓	(214)
	Papaverine	VPA-exposed rat	Protein level: BDNF↑, synapsin-IIa↑, DCX↑, pCREB↑, IL-6↓, TNF-α↓, IL-10↑, GSH↑, TBARS↓	(215)
	Rolipram	*Fmr1* mutant mouse	Protein level: GSK-3β↑, pS202-Tau/Tau↓, Tau↑ mTOR↑	(255)
Selective serotonin reuptake inhibitor	Sertraline	*Cdkl5* mutant mouse	Number of doublecortin-positive granule neurons↑, dendritic length and spine density of granule neurons↑, PSD95↑	(256)
	Fluoxetine	*Nlgn3* mutant mouse	Neurogenesis↑	(257)
		*Ube3a* mutant mouse	Number of parvalbumin-positive interneurons↑ Protein level: glucocorticod receptor↑, SGK1↑, BDNF↑, FKBP5↑	(129)
		*Fmr1* mutant mouse	Cell proliferation↔ Protein level: BDNF↔, TrkB↔	(105)
mTOR inhibitor	Rapamycin	*Pten* mutant mouse	nuclear diameter↓, dentate gyrus hypertrophy↓ Protein level: p-Ser235/236–S6 (mTORC1 activity)↓, p-AKT-S473↓	(258)
		VPA-exposed rat	Number of autophagosome↑, cell apoptosis↓ Protein level: LC3 II↑, LC3 I↓, p62↓, Bcl-2↑, p53↓, p-PI3K↓, p-AKT↓, p-S6↓	(220)
			Cell apoptosis↓ Protein level: Bcl-2↑, BDNF↑	(221)
Na-K-Cl cotransporter inhibitor	Bumetanide	VPA-exposed rat	Cl^–^ level↓	(259)
		*Fmr1* mutant mouse	Cl^–^ level↓	(259)
mGluR5 antagonist	MPEP	BTBR mouse	Protein level: p-ERK1/2↓	(242)
	LY341495	*Fmr1* mutant mouse	GSK-3β↓, pS202-Tau/Tau↓,	(255)
	Mavoglurant	*Fmr1* mutant mouse	Dendritic spine length↓	(89)
NMDAR antagonist	Agmatine	VPA-exposed rat	Protein level: p-ERK1/2↓	(260)
	Dextromethorphan	VPA-exposed rat	Protein level: NMDA↓, p-ERK1/2↓ Gene expression: NMDA↓, p-ERK1/2↓	(200)
	Ketamine	VPA-exposed rat	Gene expression: Pten↑, Psd95↔, Glur1↔, Synapsin1↔, Rab3d↔, Vamp3↔	(222)
		*MeCP2* mutant rat	Gene expression: Psd95↑, Glur1↑, Pten↔, Synapsin1↔, Rab3d↔, Vamp3↔	(222)
	Memantine	VPA-exposed rat	Number of intact neurons↑, Number of neurofibrillary tangles↓, Nissl’s granules optical density↑ Protein level: glutamate↓, GABA↑, BDNF↑, Caspase-3↓, Bax↓, Bcl-2↑, GFAP↓, CREB↔, p-CREB↔ Gene expression: Glt-1↑	(201)
Histamine H3R antagonist	E100	VPA-exposed mice	Protein level: IL-6↓, IL-1β↓, TNF-α↓, TGF-β↓, NF-κB p65↓, iNOS↓, COX-2↓	(216)
	ST-713	BTBR mouse	Protein level: ERK↓, p38↓, JNK↓, IL-6↓, IL-1β↓, TNF-α↓, histamine↑, dopamine↑	(248)
Insulin-like growth factor	Insulin-like growth factor 2	BTBR mouse	Protein level: mTOR↓, p-mTOR↓, p-S6K↓, p-AMPK↓, ULK1↓, p-ULK1↓	(261)
Aerobic exercise	Wheel running	MIA mice	Density of mossy fiber synapses↓, microglial synapse engulfment↑	(229)
		*Cdkl5* mutant mouse	Neurogenesis↑, BrdU-positive cells↑, size and density of AIF-1-positive microglial cells↓, number of immature spine↓, number of mature spine↑ Protein level: BDNF↑	(262)
		*Fmr1* mutant mouse	Cell proliferation↑, BrdU positive cells↑	(263)
	Treadmill running	VPA-exposed rat	Neurogenesis↑, Protein level: Reelin↑, PI3K↑, p-Akt↑, p-ERK1/2↑	(204)
	Swimming	*Shank3* mutant rat	Dendritic spine density↑, number of dendritic branch↑	(152)
		VPA-exposed mouse	Protein level: IL-6↓, TNF-α↓, IFN-γ↓, IL-17↓	(208)
Environmental enrichment	Maternal stimulation	*Fmr1* mutant mouse	Number of filopodia-like spines↓, number of mature thin and mushroom spines↑	(90)
	Housing condition enrichment	*Fmr1* mutant mouse	Gene expression (transcriptome analysis): Bdnf↑, Mef2c↑, Gabrg2↑, Drd1↑, Nefm↑, Prkce↑, Ncam2↑, Igsf9b↓ Protein level: BDNF↑, p-TrkB↑, p-PLCγ1↑, p-CaMKII↑	(91)
		VPA-exposed mice	Dendritic spine density↑ Gene expression: Bdnf↑, Psd95↑, Shank2↑	(209)
		*Ube3a* mutant mouse	Number of PV-positive GABAergic neurons↑ Protein level: GR↑, BDNF↑, pThr286CaMKIIα↓	(264)
		MIA rat	Gene expression: Bdnf↑	(265)

The direction of the arrows indicates increase (↑), decrease (↓), or no change (↔) compared to controls.

### 4.1 Hormones

Oxytocin has been proposed as a possible therapeutic agent for ASD due to its potent regulation of mammalian social behavior. The injection of oxytocin into the left lateral ventricle specifically improved the long-term social recognition memory and LTP at hippocampal synapses in Shank3-deficient rats (147). *In vivo*, subcutaneous oxytocin injections induced upregulation of hippocampal postsynaptic proteins PSD95 and Nlgn3 of Shank3 deficient mice (251). In rats exposed to VPA prenatally, chronic intranasal oxytocin administration rescued ASD-like behaviors including learning and memory impairments (198), and enhanced the expression of multiple genes in the hippocampus linked to synaptic function, learning, memory, and neurodevelopment (252). Erythropoietin, a glycoprotein hormone, has recently been reported to inhibit the astrogliosis in the hippocampal CA1 subfield in both LPS (227) and VPA (266) induced rat models of ASD, contributing to the enhanced learning and memory task performance (227). A preliminary study in VPA-treated rats has shown that melatonin treatment restored hippocampal CaMKII/PKA/PKC phosphorylation and LTP reduction, which might correlate with amelioration of hippocampus-dependent memory and learning skills (253).

### 4.2 Vitamins and minerals

Recent RNA sequencing research has found that vitamin A supplementation significantly alleviated VPA-induced anxiety behaviors, possibly by regulating lncRNA-mRNA co-expression networks (particularly lncRNA NONRATT021475.2 and Desert hedgehog gene) in the hippocampus of ASD rats (254). Gestational B-vitamin supplementation (vitamin B6, folic acid, and vitamin B12) has the potential to mitigate PM2.5-induced spatial learning and memory defects in mice offspring by ameliorating hippocampal inflammation, oxidative stress, mitochondrial damage, neuronal apoptosis, and synaptic dysfunction (236). Likewise, using solely folic acid rescued hippocampal neuron death and spatial learning and memory impairments in BTBR mice as it suppressed oxidative stress, inflammation, and ferroptosis in the hippocampus (240). Regarding mineral supplementation, selenium has a protective effect on the hippocampus of BTBR mice, with a comparable mechanism to that of folic acid (241). A six-week zinc water supplementation led to a decrease in anxiety-like behavior and seizure susceptibility in BTBR mice. This effect may be related to the restoration of neural progenitor cell proliferation and excitation/inhibition balance in the hippocampus (250). Lithium treatment can rescue olfactory-based learning and memory defects in drosophila fragile X model, and increase the cAMP signaling by inhibiting GSK-3β activity in the hippocampus of fragile X mice (255). In addition, lithium exerts an anti-inflammatory role probably by reducing microglial activation and inflammatory cytokine release and increasing levels of H3K9 acetylation in the hippocampus of VPA-exposed rats, with beneficial implications for improving social memory and anxiety levels (211).

### 4.3 Atypical antipsychotic drugs

The third-generation, atypical antipsychotic drugs aripiprazole, and risperidone are the only medications approved by the American FDA for ASD treatment. Data from several studies suggest that chronic treatments with aripiprazole attenuated VPA-induced visual recognition memory (207) and spatial learning and memory (201) impairments in mouse/rat offspring with ASD. More importantly, maternal treatment with aripiprazole prevents working memory deficits and hippocampal cell death in juvenile mice exposed to VPA during the prenatal period (210). Chronic administrations of risperidone improved VPA-induced memory impairment and reductions in hippocampal dendritic spine density, but there was no improvement with acute administrations (207). In addition, risperidone impeded hippocampal glutamate excitotoxicity in the VPA rat model of ASD, ultimately promoting neuronal survival (219). Similarly, the antipsychotic olanzapine can alleviate VPA-induced impairments in recognition and spatial memory by mitigating neuroplastic alterations in the hippocampus, including neuronal hypotrophy, reduced spine density, and elongated dendritic length (203).

### 4.4 Phosphodiesterase inhibitors

The phosphodiesterase class of enzymes is responsible for the degradation of cAMP, which affects various neurobiological processes from neuroinflammation to learning and memory formation (267). For instance, ibudilast, a phosphodiesterase-4 inhibitor, was found to elevate levels of oxidative stress markers (SOD, GSH, CAT) and lower levels of pro-inflammatory markers (IL-1β, TNF-α, IL-6) in the hippocampus of VPA exposed rats. Meanwhile, these ASD rats administered with two doses of ibudilast showed significantly reduced deficits in learning/memory and anxious behaviors (199). In the same way, Luhach and colleagues have demonstrated that vinpocetine, cilostazol, and papaverine, all of which are phosphodiesterase inhibitors, positively influenced neurogenesis, neuronal survival, synaptic transmission, neuronal transcription, neuronal inflammation, and neuronal oxidative stress in the hippocampus of VPA-exposed rat models (213–215). Both rolipram (phosphodiesterase-4 inhibitor) and BAY-60-7550 (phosphodiesterase-2 inhibitor) treatment abrogated the exaggerated hippocampal mGluR-LTD observed in fragile X mice, which can be attributed to significantly increased cAMP levels (88, 255, 268). In like manner, rolipram treatment rescued the LTP of hippocampal CA1 neurons to a significant level in Rett syndrome mice (269). Lastly, rolipram rescued olfactory-based long-term memory defects of drosophila fragile X models (255, 268).

### 4.5 Selective serotonin reuptake inhibitors

Sertraline and fluoxetine function as primary selective serotonin reuptake inhibitors, impeding 5-hydroxytryptamine uptake into presynaptic vesicles from the synaptic cleft in the central nervous system. Chronic treatment with sertraline improved autistic-like features in *Cdkl5* KO mice, including hippocampus-dependent spatial learning and memory deficiency. This positive behavioral effect was associated with restored neuronal survival, dendritic development, and synaptic connectivity in the dentate gyrus and CA1 pyramidal neurons (256). Recent evidence suggests that fluoxetine can ameliorate social behavior in *Nlgn3*-KO mice, at least in part, by promoting adult hippocampal neurogenesis (257). More typically, long-term fluoxetine treatment normalizes hippocampal parvalbumin-positive interneurons number and glucocorticoid signaling of Angelman syndrome model mice, which is important for the restoration of anxiety-like behaviors (129). On the contrary, however, *Fmr1* KO mice after fluoxetine treatment have reduced anxiety but enhanced explorative activity during the open field test, with abnormal changes of BDNF/TrkB signaling in the hippocampus (105). Activation with selective serotonin reuptake inhibitors has been observed in people with fragile X syndrome, which can manifest as mood changes and disinhibited behavior (270). What calls for special attention is the subsequent risk of ASD with exposure to selective serotonin reuptake inhibitors during pregnancy. Recent findings underscored that maternal fluoxetine exposure impaired hippocampal LTP, spatial discrimination, and spatial learning in adult offspring (271), accompanied by decreased hippocampal neurogenesis and hippocampal IL-10, IFN-γ and IL-13 levels (272).

### 4.6 mTOR inhibitors

Dysregulation of mTOR signaling is strongly associated with ASD, and the inhibition of mTOR can prevent the binding of mTOR with other protein components and reduce mTOR phosphorylation (273). For example, rapamycin, as a prominent mTOR inhibitor, has been shown to effectively block the hippocampal mTORC1 signaling of Pten mutant mice (258). Besides, rapamycin plays a crucial role in promoting autophagy (220) and decreasing apoptosis (220, 221) in the hippocampus of VPA-induced neonatal rats of ASD, thereby improving learning and memory ability (221). As discussed earlier, tuberous sclerosis complex models had increased hippocampal neuron volume, blood flow, and oxygen consumption. Correspondingly, the administration of rapamycin has been reported to lower cerebral blood flow and oxygen consumption in hippocampal regions of the Tsc2 mutant rat, potentially by downregulating Akt signals (146). This also accords with previous observations, which showed that pharmacological inhibition of mTORC suppressed granule cell hypertrophy in Pten mutant mice (258) and inhibited the PI3K/AKT/mTOR signaling pathway in VPA-induced rats (220).

### 4.7 Na-K-Cl cotransporter inhibitors

Bumetanide, a loop diuretic that inhibits the Na-K-Cl cotransporter, has been reported to improve core symptoms of ASD in children over recent years (274). In VPA-treated rats, even a brief maternal bumetanide treatment can prevent hippocampal overgrowth in their offspring (275). In addition, maternal pretreatment with bumetanide effectively restored elevated hippocampal intracellular chloride levels, increased hippocampal excitatory GABA, and enhanced hippocampal gamma oscillations in offspring of VPA-induced rats and *Fmr1* mutant mice (259). Recently, a new selective Na-K-Cl cotransporter inhibitor called ARN23746 has been reported to improve sociability in VPA-induced mice, similar to bumetanide (276). The oral administration of torasemide, a diuretic that also acts as a Na-K-Cl cotransporter inhibitor, has the potential to enhance neuronal viability and reduce astrogliosis in the hippocampus of ASD rat models (277).

### 4.8 mGluR5 antagonists

The dysregulated signaling mediated via mGluR5 contributes to the pathophysiology of ASD since it acts as a crucial regulator of excitatory and inhibitory signaling in the hippocampus (278). After treatment with the mGluR5 antagonist MPEP, epileptiform activity and ERK signaling in the hippocampal slices of Tsc2 mutant mice can be suppressed. The blocked mGluR-LTD in the CA3 hippocampus via antagonism of mGluR5 also improved reversal learning performance between these mice (142). Similarly, for the BTBR mouse model, MPEP treatment facilitated hippocampus-dependent object location memory and decreased synaptic p-ERK1/2 levels (242). Like lithium and rolipram, fragile X syndrome flies treated with mGluR5 antagonist LY341495 demonstrated an enhanced long-term memory paradigm compared to controls (255). Long-term antagonism of mGluR5 also rescued immature spine phenotype (89) and decreased GSK-3β activity (255) in the hippocampus of *Fmr1*-KO mice.

### 4.9 NMDAR antagonists

NMDAR-mediated excitation and inhibition imbalance is one of the primary theories explaining the neurotoxicity in ASD. Data from several studies on rats exposed to VPA indicate that the NMDAR antagonist, agmatine (260) and dextromethorphan (200), normalize the overly active ERK1/2 phosphorylation in the hippocampus, which serves as an indicative marker of hippocampal hyperexcitability state. Besides, spatial memory and learning deficits induced by *Fmr1*-KO and VPA were mitigated following agmatine (100) and dextromethorphan (200) administration, respectively. For both rats exposed to VPA and those with a *Mecp2*-KO, the administration of ketamine produces a positive effect on their autistic-like behaviors by improving synaptic molecule levels in the dentate gyrus of the hippocampus (222). Memantine, a non-competitive antagonist of NMDAR, was reported to alleviate anxiety and improve learning and memory deficits in VPA-exposed rats, which could be mediated via the restoration of hippocampal GABA/glutamate balance and inhibition of hippocampal neurofibrillary tangles formation and neuronal apoptosis (201).

### 4.10 Histamine H3R antagonists

The histamine H3R, as a presynaptic autoreceptor can regulate the production and release of histamine as well as numerous brain neurotransmitters like dopamine and acetylcholine (279). Thus, selective H3R antagonists can improve the cognitive impairment in ASD. It is evident from the observation that the administration of histamine H3R antagonists ST-2223 (247) and ST-713 (248) significantly elevated the levels of histamine, dopamine, and acetylcholine in the hippocampal tissue of BTBR mice with anxiolytic-like effects. Administration of ciproxifan could improve the VPA-induced LTP decline in the CA1 area and hippocampus-dependent learning and memory capacity (202). Moreover, the histamine H3R antagonist E100 mitigates VPA-induced hippocampal inflammation by reducing the levels of IL-6, IL-1β, TNF-α, and TGF-β and by suppressing the expression of NF-κB, iNOS, and COX-2 (216).

### 4.11 Insulin-like growth factor

The insulin-like growth factor (IGF) system comprising two activating ligands (IGF-1 and IGF-2) greatly impacts the development of the central nervous system. It has been discovered that levels of IGF-1 are reduced in the hippocampus of Rett syndrome mouse model (280). The active peptide derivative of IGF-1 can cross the blood-brain barrier and rescue Rett syndrome symptoms in *MeCP2* mutant mice (281). In addition, daily intraperitoneal injections of IGF-1 for 2 weeks reversed deficits in hippocampal LTP in *Shank3*-deficient mice (282). Previous data revealed that *Nlgn3* KO mice treated with IGF-2 fully recovered the social novelty discrimination. This effect was not accompanied by any alteration in spontaneous glutamatergic synaptic transmission within the CA2 region, but rather by enhanced CA2 neuronal excitability (283). Similarly, the administration of IGF-2 ameliorated social interaction deficits in BTBR mice and enhanced their social novelty memory via hippocampal IGF-2 receptor (261). In the Angelman syndrome mouse model, the impaired contextual and recognition memories as well as working memory deficits were restored following subcutaneous injection of IGF-2 (284).

### 4.12 Aerobic exercises

The beneficial effects on memory functions of aerobic exercise, as a non-pharmacological intervention, have been well-documented in individuals with ASD (285). Compared to sedentary controls, both *Cdk*l5 KO mice (262) and VPA-induced rats (204) after one month of wheel/treadmill running showed increased hippocampal neurogenesis, improved memory performance, and reduced anxious and impulsive behaviors. One-month voluntary wheel running not only can decrease hippocampal microglia overactivation in *Cdkl5* KO mice (262), but also stimulate microglia-mediated engulfment of surplus synapses in the granule cell axons observed in MIA mice (229). On the other hand, however, high-intensity exercise may produce negative effects on ASD symptoms. For instance, two-month treadmill training with relatively high speed led to impaired social memory in ASD rats with mercury exposure (286). Voluntary running for seven days increased cell proliferation in the hippocampal dentate gyrus of *Fmr1* KO mice, but this effect was not observed when running for 28 days (263). Swimming is another beneficial aerobic exercise that has been shown to increase hippocampal gray matter volume and dendritic spine density in *Shank3*-KO rats (152) and to prevent hippocampal neuroinflammation in VPA-exposed mice (208). It also contributes to improvements in spatial, working, social, and visual recognition memory (152, 208).

### 4.13 Environmental enrichment

Environmental enrichment is a novel living condition with increased social exploration opportunities and sensory, cognitive, and motor stimulations, which positively affect the hippocampus-dependent learning, memory, and anxiety behavior of children with ASD (287). Living with the mother and an additional non-lactating female (enhanced maternal stimulation) can rescue the spatial and contextual fear memory deficits displayed in adulthood by *Fmr1*-KO mice (90). In addition to this “social environmental enrichment”, enrichment in housing conditions (running wheel, toys, tunnels, ladders, etc.) also ameliorates impaired anxiety-like behavior (91, 209, 264), visual recognition memory (209, 264), spatial learning and memory (91, 265), and fear memory (91), potentially by acting on the hippocampal BDNF/TrkB-PLCγ1-CaMKII pathway (91, 209, 264, 265), as observed in *Fmr1*-KO mice (91), VPA-exposed mice (209), *Ube3a*-KO mice (264), and MIA rats (265).

## 5 Discussion

In summary, hippocampal involvement in the pathophysiology of ASD is now an acquired knowledge. While the majority of current structural and functional neuroimaging studies concentrate on the social brain networks in ASD, hippocampal formation should not be overlooked as it plays a crucial role in higher non-social cognitive functions that are also significantly impaired in most ASD individuals. Numerous lines of clinical evidence on hippocampal volume, morphology, blood flow, metabolism, and functional connectivity seem to converge toward the hypothesis of a hippocampal neurofunctional deficit in ASD, which concerns learning, memory, language ability, emotional regulation, and cognitive map creation. Furthermore, researches based on different ASD animal models are rapidly enhancing our understanding of the neural mechanisms underlying hippocampus-dependent behavioral deficits. In general, typical hallmarks of hippocampal deviations in ASD involve impairments in neurogenesis, dendritic morphology, neuronal viability, neuronal excitation/inhibition balance, LTP, and LTD. Results from the recent therapeutic approaches for ASD are encouraging, since some behavioral alterations such as learning and memory deficits, could be reversed even when treatment was performed on adult mice/rats, potentially by influencing the structure and function of the hippocampus. By targeting therapy at the site of hippocampal pathology, more effective pharmacological and non-pharmacological approaches may be developed in the future.

Despite these promising results, several questions remain unanswered at present. Firstly, as we noted above it is necessary to further confirm whether hippocampal abnormalities, particularly changes in volume, are present in all individuals with ASD or only in a specific subgroup. To date, comparable anatomical and morphological alterations have been detected in the hippocampus of individuals with ASD and ASD animal models, but human postmortem analysis is still limited by the relatively small numbers of individual brains. Besides, the developmental profile of hippocampal pathology in ASD remains unknown. It cannot be discounted that alternation in hippocampal structure may be secondary due to the disease process, the emotional stress, or the various treatments of ASD. Integrating the neuropathology of the hippocampus in ASD individuals with the aetiology and pathophysiology of ASD is a major challenge for the coming years. Other issues to be addressed involve ascertaining the precision of diagnosing ASD by detecting hippocampal lesions and determining the significance of hippocampal pathology compared to other affected regions. Finally, it is beyond dispute that individuals with ASD exhibit whole-brain functional connectivity deficits, and it is not possible to simply attribute the symptoms of ASD to specific regions of the brain. More specifically, as discussed above atypical connectivity at the local network level between the hippocampus and other brain regions could potentially account for impaired non-social behaviors in ASD. The utilization of multimodal neuroimaging data, such as CT, MRI, fNIRS, EEG, MEG, etc., is progressively increasing in both scientific research and clinical settings nowadays. In future investigations, it is hoped that this multilevel approach will provide insight into the neural circuits behind the hippocampus-dependent functional deficits in those with ASD, which can in turn elucidate the developmental mechanisms underlying hippocampal pathology in ASD.

## Author contributions

JL: Writing – original draft, Conceptualization. HL: Writing – original draft. YL: Software, Visualization, Writing – review & editing. XL: Software, Visualization, Writing – review & editing. ZT: Software, Visualization, Writing – review & editing. KH: Software, Visualization, Writing – review & editing. JC: Writing – review & editing, Software, Visualization. HZ: Conceptualization, Funding acquisition, Supervision, Writing – original draft.

## References

[1] Talantseva OI Romanova RS Shurdova EM Dolgorukova TA Sologub PS Titova OS. The global prevalence of autism spectrum disorder: A three-level meta-analysis. Front Psychiatry. (2023) 14:1071181. doi: 10.3389/fpsyt.2023.1071181 36846240 PMC9947250

[2] Lord C Brugha TS Charman T Cusack J Dumas G Frazier T. Autism spectrum disorder. Nat Rev Dis Primers. (2020) 6:5. doi: 10.1038/s41572-019-0138-4 31949163 PMC8900942

[3] Dawson G Webb S Schellenberg GD Dager S Friedman S Aylward E. Defining the broader phenotype of autism: genetic, brain, and behavioral perspectives. Dev Psychopathol. (2002) 14:581–611. doi: 10.1017/S0954579402003103 12349875

[4] Smith AD. Spatial navigation in autism spectrum disorders: a critical review. Front Psychol. (2015) 6:31. doi: 10.3389/fpsyg.2015.00031 25667579 PMC4304163

[5] Williams DL Goldstein G Minshew NJ. Impaired memory for faces and social scenes in autism: clinical implications of memory dysfunction. Arch Clin Neuropsychol. (2005) 20:1–15. doi: 10.1016/j.acn.2002.08.001 15620811

[6] Wang Y Zhang YB Liu LL Cui JF Wang J Shum DH. A meta-analysis of working memory impairments in autism spectrum disorders. Neuropsychol Rev. (2017) 27:46–61. doi: 10.1007/s11065-016-9336-y 28102493

[7] O'Brien G Pearson J. Autism and learning disability. Autism. (2004) 8:125–40. doi: 10.1177/1362361304042718 15165430

[8] Luyster R Lord C. Word learning in children with autism spectrum disorders. Dev Psychol. (2009) 45:1774–86. doi: 10.1037/a0016223 PMC3035482 19899931

[9] Norrelgen F Fernell E Eriksson M Hedvall Å Persson C Sjölin M. Children with autism spectrum disorders who do not develop phrase speech in the preschool years. Autism. (2015) 19:934–43. doi: 10.1177/1362361314556782 25488002

[10] Kirsch AC Huebner ARS Mehta SQ Howie FR Weaver AL Myers SM. Association of comorbid mood and anxiety disorders with autism spectrum disorder. JAMA Pediatr. (2020) 174:63–70. doi: 10.1001/jamapediatrics.2019.4368 31790555 PMC6902186

[11] Lind SE Williams DM Raber J Peel A Bowler DM. Spatial navigation impairments among intellectually high-functioning adults with autism spectrum disorder: exploring relations with theory of mind, episodic memory, and episodic future thinking. J Abnorm Psychol. (2013) 122:1189–99. doi: 10.1037/a0034819 PMC3906800 24364620

[12] Lind SE Bowler DM Raber J. Spatial navigation, episodic memory, episodic future thinking, and theory of mind in children with autism spectrum disorder: evidence for impairments in mental simulation? Front Psychol. (2014) 5:1411. doi: 10.3389/fpsyg.2014.01411 25538661 PMC4256988

[13] Pellicano E Smith AD Cristino F Hood BM Briscoe J Gilchrist ID. Children with autism are neither systematic nor optimal foragers. Proc Natl Acad Sci USA. (2011) 108:421–6. doi: 10.1073/pnas.1014076108 PMC3017188 21173235

[14] Lisman J Buzsáki G Eichenbaum H Nadel L Ranganath C Redish AD. Viewpoints: how the hippocampus contributes to memory, navigation and cognition. Nat Neurosci. (2017) 20:1434–47. doi: 10.1038/nn.4661 PMC5943637 29073641

[15] Izquierdo I. The hippocampus and learning. Prog Neurobiol. (1975) 5:37–75. doi: 10.1016/0301-0082(75)90007-6 830080

[16] Covington NV Duff MC. Expanding the language network: direct contributions from the hippocampus. Trends Cognit Sci. (2016) 20:869–70. doi: 10.1016/j.tics.2016.10.006 PMC5345935 27814958

[17] Qasim SE Mohan UR Stein JM Jacobs J. Neuronal activity in the human amygdala and hippocampus enhances emotional memory encoding. Nat Hum Behav. (2023) 7:754–64. doi: 10.1038/s41562-022-01502-8 PMC11243592 36646837

[18] Hogeveen J Krug MK Geddert RM Ragland JD Solomon M. Compensatory hippocampal recruitment supports preserved episodic memory in autism spectrum disorder. Biol Psychiatry Cognit Neurosci Neuroimaging. (2020) 5:97–109. doi: 10.1016/j.bpsc.2019.08.009 31676207 PMC6954323

[19] Ring M Derwent CLT Gaigg SB Bowler DM. Structural learning difficulties implicate altered hippocampal functioning in adults with autism spectrum disorder. J Abnorm Psychol. (2017) 126:793–804. doi: 10.1037/abn0000277 28557507

[20] Boucher J Cowell P Howard M Broks P Farrant A Roberts N. A combined clinical, neuropsychological, and neuroanatomical study of adults with high functioning autism. Cognit Neuropsychiatry. (2005) 10:165–213. doi: 10.1080/13546800444000038 16571459

[21] Endo T Shioiri T Kitamura H Kimura T Endo S Masuzawa N. Altered chemical metabolites in the amygdala-hippocampus region contribute to autistic symptoms of autism spectrum disorders. Biol Psychiatry. (2007) 62:1030–7. doi: 10.1016/j.biopsych.2007.05.015 17631869

[22] Allsop SA Vander Weele CM Wichmann R Tye KM. Optogenetic insights on the relationship between anxiety-related behaviors and social deficits. Front Behav Neurosci. (2014) 8:241. doi: 10.3389/fnbeh.2014.00241 25076878 PMC4099964

[23] Destrieux C Bourry D Velut S. Surgical anatomy of the hippocampus. Neurochirurgie. (2013) 59:149–58. doi: 10.1016/j.neuchi.2013.08.003 24183470

[24] El-Falougy H Benuska J. History, anatomical nomenclature, comparative anatomy and functions of the hippocampal formation. Bratisl Lek Listy. (2006) 107:103–6. 16796134

[25] Rajmohan V Mohandas E. The limbic system. Indian J Psychiatry. (2007) 49:132–9. doi: 10.4103/0019-5545.33264 PMC2917081 20711399

[26] Weininger J Roman E Tierney P Barry D Gallagher H Murphy P. Papez's forgotten tract: 80 years of unreconciled findings concerning the thalamocingulate tract. Front Neuroanat. (2019) 13:14. doi: 10.3389/fnana.2019.00014 30833890 PMC6388660

[27] Gindes L Weissmann-Brenner A Weisz B Zajicek M Geffen KT Achiron R. Identification of the fetal hippocampus and fornix and role of 3-dimensional sonography. J Ultrasound Med. (2011) 30:1613–8. doi: 10.7863/jum.2011.30.12.1613 22123994

[28] Utsunomiya H Takano K Okazaki M Mitsudome A. Development of the temporal lobe in infants and children: analysis by MR-based volumetry. AJNR Am J Neuroradiol. (1999) 20:717–23. PMC7056017 10319988

[29] Hazlett HC Gu H Munsell BC Kim SH Styner M Wolff JJ. Early brain development in infants at high risk for autism spectrum disorder. Nature. (2017) 542:348–51. doi: 10.1038/nature21369 PMC5336143 28202961

[30] Mandell DS Novak MM Zubritsky CD. Factors associated with age of diagnosis among children with autism spectrum disorders. Pediatrics. (2005) 116:1480–6. doi: 10.1542/peds.2005-0185 PMC2861294 16322174

[31] Banker SM Gu X Schiller D Foss-Feig JH. Hippocampal contributions to social and cognitive deficits in autism spectrum disorder. Trends Neurosci. (2021) 44:793–807. doi: 10.1016/j.tins.2021.08.005 34521563 PMC8484056

[32] Eilam-Stock T Wu T Spagna A Egan LJ Fan J. Neuroanatomical alterations in high-functioning adults with autism spectrum disorder. Front Neurosci. (2016) 10:237. doi: 10.3389/fnins.2016.00237 27313505 PMC4889574

[33] Arutiunian V Davydova E Pereverzeva D Sorokin A Tyushkevich S Mamokhina U. Reduced grey matter volume of amygdala and hippocampus is associated with the severity of autistic symptoms and language abilities in school-aged children with Autism Spectrum Disorder: an exploratory study. Brain Struct Funct. (2023) 228:1573–9. doi: 10.1007/s00429-023-02660-9 37302090

[34] Groen W Teluij M Buitelaar J Tendolkar I. Amygdala and hippocampus enlargement during adolescence in autism. J Am Acad Child Adolesc Psychiatry. (2010) 49:552–60. doi: 10.1016/j.jaac.2009.12.023 20494265

[35] Sussman D Leung RC Vogan VM Lee W Trelle S Lin S. The autism puzzle: Diffuse but not pervasive neuroanatomical abnormalities in children with ASD. NeuroImage Clin. (2015) 8:170–9. doi: 10.1016/j.nicl.2015.04.008 PMC4473820 26106541

[36] Aylward EH Minshew NJ Goldstein G Honeycutt NA Augustine AM Yates KO. MRI volumes of amygdala and hippocampus in non-mentally retarded autistic adolescents and adults. Neurology. (1999) 53:2145–50. doi: 10.1212/WNL.53.9.2145 10599796

[37] Nees F Banaschewski T Bokde ALW Desrivières S Grigis A Garavan H. Global and regional structural differences and prediction of autistic traits during adolescence. Brain Sci. (2022) 12:1187. doi: 10.3390/brainsci12091187 36138923 PMC9496772

[38] Pagni BA Walsh MJM Ofori E Chen K Sullivan G Alvar J. Effects of age on the hippocampus and verbal memory in adults with autism spectrum disorder: Longitudinal versus cross-sectional findings. Autism Res. (2022) 15:1810–23. doi: 10.1002/aur.2797 PMC9561078 36053945

[39] Lee JK Nordahl CW Amaral DG Lee A Solomon M Ghetti S. Assessing hippocampal development and language in early childhood: Evidence from a new application of the Automatic Segmentation Adapter Tool. Hum Brain Mapp. (2015) 36:4483–96. doi: 10.1002/hbm.22931 PMC4913550 26279309

[40] Nicolson R DeVito TJ Vidal CN Sui Y Hayashi KM Drost DJ. Detection and mapping of hippocampal abnormalities in autism. Psychiatry Res. (2006) 148:11–21. doi: 10.1016/j.pscychresns.2006.02.005 17056234

[41] Sacco R Gabriele S Persico AM. Head circumference and brain size in autism spectrum disorder: A systematic review and meta-analysis. Psychiatry Res. (2015) 234:239–51. doi: 10.1016/j.pscychresns.2015.08.016 26456415

[42] Maier S Tebartz van Elst L Beier D Ebert D Fangmeier T Radtke M. Increased hippocampal volumes in adults with high functioning autism spectrum disorder and an IQ>100: A manual morphometric study. Psychiatry Res. (2015) 234:152–5. doi: 10.1016/j.pscychresns.2015.08.002 26337007

[43] Xu Q Zuo C Liao S Long Y Wang Y. Abnormal development pattern of the amygdala and hippocampus from childhood to adulthood with autism. J Clin Neurosci. (2020) 78:327–32. doi: 10.1016/j.jocn.2020.03.049 32593622

[44] Markram K Markram H. The intense world theory - a unifying theory of the neurobiology of autism. Front Hum Neurosci. (2010) 4:224. doi: 10.3389/fnhum.2010.00224 21191475 PMC3010743

[45] Habata K Cheong Y Kamiya T Shiotsu D Omori IM Okazawa H. Relationship between sensory characteristics and cortical thickness/volume in autism spectrum disorders. Transl Psychiatry. (2021) 11:616. doi: 10.1038/s41398-021-01743-7 34873147 PMC8648722

[46] Schumann CM Hamstra J Goodlin-Jones BL Lotspeich LJ Kwon H Buonocore MH. The amygdala is enlarged in children but not adolescents with autism; the hippocampus is enlarged at all ages. J Neurosci. (2004) 24:6392–401. doi: 10.1523/JNEUROSCI.1297-04.2004 PMC6729537 15254095

[47] Murphy CM Deeley Q Daly EM Ecker C O'Brien FM Hallahan B. Anatomy and aging of the amygdala and hippocampus in autism spectrum disorder: an in *vivo* magnetic resonance imaging study of Asperger syndrome. Autism Res. (2012) 5:3–12. doi: 10.1002/aur.227 21948742

[48] Dager SR Wang L Friedman SD Shaw DW Constantino JN Artru AA. Shape mapping of the hippocampus in young children with autism spectrum disorder. AJNR Am J Neuroradiol. (2007) 28:672–7. PMC7977363 17416819

[49] Zuo C Wang D Tao F Wang Y. Changes in the development of subcortical structures in autism spectrum disorder. Neuroreport. (2019) 30:1062–7. doi: 10.1097/WNR.0000000000001300 31464839

[50] Reinhardt VP Iosif AM Libero L Heath B Rogers SJ Ferrer E. Understanding hippocampal development in young children with autism spectrum disorder. J Am Acad Child Adolesc Psychiatry. (2020) 59:1069–79. doi: 10.1016/j.jaac.2019.08.008 PMC9940822 31449875

[51] Richards R Greimel E Kliemann D Koerte IK Schulte-Körne G Reuter M. Increased hippocampal shape asymmetry and volumetric ventricular asymmetry in autism spectrum disorder. NeuroImage Clin. (2020) 26:102207. doi: 10.1016/j.nicl.2020.102207 32092683 PMC7037573

[52] Saitoh O Karns CM Courchesne E. Development of the hippocampal formation from 2 to 42 years: MRI evidence of smaller area dentata in autism. Brain. (2001) 124:1317–24. doi: 10.1093/brain/124.7.1317 11408327

[53] Rojas DC Smith JA Benkers TL Camou SL Reite ML Rogers SJ. Hippocampus and amygdala volumes in parents of children with autistic disorder. Am J Psychiatry. (2004) 161:2038–44. doi: 10.1176/appi.ajp.161.11.2038 15514404

[54] Ferreira D Hansson O Barroso J Molina Y MaChado A Hernández-Cabrera JA. The interactive effect of demographic and clinical factors on hippocampal volume: A multicohort study on 1958 cognitively normal individuals. Hippocampus. (2017) 27:653–67. doi: 10.1002/hipo.22721 28394034

[55] Kemper TL Bauman ML. The contribution of neuropathologic studies to the understanding of autism. Neurol Clin. (1993) 11:175–87. doi: 10.1016/S0733-8619(18)30176-2 8441369

[56] Raymond GV Bauman ML Kemper TL. Hippocampus in autism: a Golgi analysis. Acta Neuropathol. (1996) 91:117–9. doi: 10.1007/s004010050401 8773156

[57] Bailey A Luthert P Dean A Harding B Janota I Montgomery M. A clinicopathological study of autism. Brain. (1998) 121:889–905. doi: 10.1093/brain/121.5.889 9619192

[58] Lawrence YA Kemper TL Bauman ML Blatt GJ. Parvalbumin-, calbindin-, and calretinin-immunoreactive hippocampal interneuron density in autism. Acta Neurol Scand. (2010) 121:99–108. doi: 10.1111/j.1600-0404.2009.01234.x 19719810

[59] Salmond CH Ashburner J Connelly A Friston KJ Gadian DG Vargha-Khadem F. The role of the medial temporal lobe in autistic spectrum disorders. Eur J Neurosci. (2005) 22:764–72. doi: 10.1111/j.1460-9568.2005.04217.x 16101758

[60] Sun F Chen Y Gao Q Zhao Z. Abnormal gray matter structure in children and adolescents with high-functioning autism spectrum disorder. Psychiatry Res Neuroimaging. (2022) 327:111564. doi: 10.1016/j.pscychresns.2022.111564 36384063

[61] Wegiel J Kuchna I Nowicki K Imaki H Wegiel J Marchi E. The neuropathology of autism: defects of neurogenesis and neuronal migration, and dysplastic changes. Acta Neuropathol. (2010) 119:755–70. doi: 10.1007/s00401-010-0655-4 PMC2869041 20198484

[62] Allebone J Kanaan R Maller J O'Brien T Mullen SA Cook M. Bilateral volume reduction in posterior hippocampus in psychosis of epilepsy. J Neurol Neurosurg Psychiatry. (2019) 90:688–94. doi: 10.1136/jnnp-2018-319396 30796132

[63] Tang S Nie L Liu X Chen Z Zhou Y Pan Z. Application of quantitative magnetic resonance imaging in the diagnosis of autism in children. Front Med. (2022) 9:818404. doi: 10.3389/fmed.2022.818404 PMC9133426 35646984

[64] Tang S Liu X Ran Q Nie L Wu L Pan Z. Application of three-dimensional pseudocontinuous arterial spin labeling perfusion imaging in the brains of children with autism. Front Neurol. (2022) 13:851430. doi: 10.3389/fneur.2022.851430 35280268 PMC8905523

[65] Tang S Liu X Nie L Chen Z Ran Q He L. Diagnosis of children with attention-deficit/hyperactivity disorder. (ADHD) comorbid autistic traits. (ATs) by applying quantitative magnetic resonance imaging techniques. Front Psychiatry. (2022) 13:1038471. doi: 10.3389/fpsyt.2022.1038471 36465303 PMC9712964

[66] Heo S Prakash RS Voss MW Erickson KI Ouyang C Sutton BP. Resting hippocampal blood flow, spatial memory and aging. Brain Res. (2010) 1315:119–27. doi: 10.1016/j.brainres.2009.12.020 PMC2822086 20026320

[67] Pagani M Manouilenko I Stone-Elander S Odh R Salmaso D Hatherly R. Brief Report: alterations in cerebral blood flow as assessed by PET/CT in adults with autism spectrum disorder with normal IQ. J Autism Dev Disord. (2012) 42:313–18. doi: 10.1007/s10803-011-1240-y 21487836

[68] Otsuka H Harada M Mori K Hisaoka S Nishitani H. Brain metabolites in the hippocampus-amygdala region and cerebellum in autism: an 1H-MR spectroscopy study. Neuroradiology. (1999) 41:517–19. doi: 10.1007/s002340050795 10450847

[69] Gabis L Huang W Azizian A DeVincent C Tudorica A Kesner-Baruch Y. 1H-magnetic resonance spectroscopy markers of cognitive and language ability in clinical subtypes of autism spectrum disorders. J Child Neurol. (2008) 23:766–74. doi: 10.1177/0883073808315423 18487520

[70] Liserre R Pinelli L Gasparotti R. MR spectroscopy in pediatric neuroradiology. Transl Pediatr. (2021) 10:1169–200. doi: 10.21037/tp-20-445 PMC8107850 34012861

[71] Page LA Daly E Schmitz N Simmons A Toal F Deeley Q. *In vivo* 1H-magnetic resonance spectroscopy study of amygdala-hippocampal and parietal regions in autism. Am J Psychiatry. (2006) 163:2189–192. doi: 10.1176/ajp.2006.163.12.2189 17151175

[72] O'Brien FM Page L O'Gorman RL Bolton P Sharma A Baird G. Maturation of limbic regions in Asperger syndrome: a preliminary study using proton magnetic resonance spectroscopy and structural magnetic resonance imaging. Psychiatry Res. (2010) 184:77–85. doi: 10.1016/j.pscychresns.2010.08.007 20952166

[73] Zeegers M van der Grond J van Daalen E Buitelaar J van Engeland H. Proton magnetic resonance spectroscopy in developmentally delayed young boys with or without autism. J Neural Transm. (2007) 114:289–95. doi: 10.1007/s00702-006-0501-y 16715207

[74] Brown MS Singel D Hepburn S Rojas DC. Increased glutamate concentration in the auditory cortex of persons with autism and first-degree relatives: a. (1)H-MRS study. Autism Res. (2013) b6:1–10. doi: 10.1002/aur.1260 PMC3580156 23166003

[75] Sokol DK Dunn DW Edwards-Brown M Feinberg J. Hydrogen proton magnetic resonance spectroscopy in autism: preliminary evidence of elevated choline/creatine ratio. J Child Neurol. (2002) 17:245–9. doi: 10.1177/088307380201700401 12088077

[76] Suzuki K Nishimura K Sugihara G Nakamura K Tsuchiya KJ Matsumoto K. Metabolite alterations in the hippocampus of high-functioning adult subjects with autism. Int J Neuropsychopharmacol. (2010) 13:529–34. doi: 10.1017/S1461145709990952 19895725

[77] Hashimoto T Yokota S Matsuzaki Y Kawashima R. Intrinsic hippocampal functional connectivity underlying rigid memory in children and adolescents with autism spectrum disorder: A case-control study. Autism. (2021) 25:1901–12. doi: 10.1177/13623613211004058 PMC8419294 33779333

[78] Liu J Chen L Chang H Rudoler J Al-Zughoul AB Kang JB. Replicable patterns of memory impairments in children with autism and their links to hyperconnected brain circuits. Biol Psychiatry Cognit Neurosci Neuroimaging. (2023) 8:1113–23. doi: 10.1016/j.bpsc.2023.05.002 PMC10646152 37196984

[79] Solomon M Ragland JD Niendam TA Lesh TA Beck JS Matter JC. Atypical learning in autism spectrum disorders: A functional magnetic resonance imaging study of transitive inference. J Am Acad Child Adolesc Psychiatry. (2015) 54:947–55. doi: 10.1016/j.jaac.2015.08.010 PMC4624100 26506585

[80] Liu J Okada NJ Cummings KK Jung J Patterson G Bookheimer SY. Emerging atypicalities in functional connectivity of language-related networks in young infants at high familial risk for ASD. Dev Cognit Neurosci. (2020) 45:100814. doi: 10.1016/j.dcn.2020.100814 32658762 PMC7341340

[81] Klapwijk ET Aghajani M Colins OF Marijnissen GM Popma A van Lang ND. Different brain responses during empathy in autism spectrum disorders versus conduct disorder and callous-unemotional traits. J Child Psychol Psychiatry. (2016) 57:737–47. doi: 10.1111/jcpp.12498 26681358

[82] Green SA Rudie JD Colich NL Wood JJ Shirinyan D Hernandez L. Overreactive brain responses to sensory stimuli in youth with autism spectrum disorders. J Am Acad Child Adolesc Psychiatry. (2013) 52:1158–72. doi: 10.1016/j.jaac.2013.08.004 PMC3820504 24157390

[83] Loveland KA Bachevalier J Pearson DA Lane DM. Fronto-limbic functioning in children and adolescents with and without autism. Neuropsychologia. (2008) 46:49–62. doi: 10.1016/j.neuropsychologia.2007.08.017 17936314 PMC2785231

[84] Mouga S Duarte IC Café C Sousa D Duque F Oliveira G. Parahippocampal deactivation and hyperactivation of central executive, saliency and social cognition networks in autism spectrum disorder. J Neurodev Disord. (2022) 14:9. doi: 10.1186/s11689-022-09417-1 35078414 PMC8903486

[85] Beaudet AL. Autism: highly heritable but not inherited. Nat Med. (2007) 13:534–6. doi: 10.1038/nm0507-534 17479094

[86] Li Z Zhu YX Gu LJ Cheng Y. Understanding autism spectrum disorders with animal models: applications, insights, and perspectives. Zool Res. (2021) 42:800–24. doi: 10.24272/j.issn.2095-8137.2021.251 PMC8645879 34755500

[87] Fyke W Velinov M. FMR1 and autism, an intriguing connection revisited. Genes. (2021) 12:1218. doi: 10.3390/genes12081218 34440392 PMC8394635

[88] Maurin T Melancia F Jarjat M Castro L Costa L Delhaye S. Involvement of phosphodiesterase 2A activity in the pathophysiology of fragile X syndrome. Cereb Cortex. (2019) 29:3241–52. doi: 10.1093/cercor/bhy192 30137253

[89] Pop AS Levenga J de Esch CE Buijsen RA Nieuwenhuizen IM Li T. Rescue of dendritic spine phenotype in Fmr1 KO mice with the mGluR5 antagonist AFQ056/Mavoglurant. Psychopharmacology. (2014) 231:1227–35. doi: 10.1007/s00213-012-2947-y 23254376

[90] Oddi D Subashi E Middei S Bellocchio L Lemaire-Mayo V Guzmán M. Early social enrichment rescues adult behavioral and brain abnormalities in a mouse model of fragile X syndrome. Neuropsychopharmacology. (2015) 40:1113–22. doi: 10.1038/npp.2014.291 PMC4367453 25348604

[91] Chen YS Zhang SM Yue CX Xiang P Li JQ Wei Z. Early environmental enrichment for autism spectrum disorder Fmr1 mice models has positive behavioral and molecular effects. Exp Neurol. (2022) 352:114033. doi: 10.1016/j.expneurol.2022.114033 35259351

[92] Lazarov O Demars MP Zhao Kda T Ali HM Grauzas V Kney A. Impaired survival of neural progenitor cells in dentate gyrus of adult mice lacking fMRP. Hippocampus. (2012) 22:1220–24. doi: 10.1002/hipo.20989 PMC3291746 22128095

[93] Braun K Segal M. FMRP involvement in formation of synapses among cultured hippocampal neurons. Cereb Cortex. (2000) 10:1045–52. doi: 10.1093/cercor/10.10.1045 11007555

[94] Klemmer P Meredith RM Holmgren CD Klychnikov OI Stahl-Zeng J Loos M. Proteomics, ultrastructure, and physiology of hippocampal synapses in a fragile X syndrome mouse model reveal presynaptic phenotype. J Biol Chem. (2011) 286:25495–504. doi: 10.1074/jbc.M110.210260 PMC3138307 21596744

[95] Zhang J Hou L Klann E Nelson DL. Altered hippocampal synaptic plasticity in the Fmr1 gene family knockout mouse models. J Neurophysiol. (2009) 101:2572–80. doi: 10.1152/jn.90558.2008 PMC2681424 19244359

[96] Wang XS Peng CZ Cai WJ Xia J Jin D Dai Y. Activity-dependent regulation of release probability at excitatory hippocampal synapses: a crucial role of fragile X mental retardation protein in neurotransmission. Eur J Neurosci. (2014) 39:1602–12. doi: 10.1111/ejn.12546 PMC4028396 24646437

[97] Yun SH Trommer BL. Fragile X mice: reduced long-term potentiation and N-Methyl-D-Aspartate receptor-mediated neurotransmission in dentate gyrus. J Neurosci Res. (2011) 89:176–82. doi: 10.1002/jnr.22546 21162125

[98] Tian Y Yang C Shang S Cai Y Deng X Zhang J. Loss of FMRP impaired hippocampal long-term plasticity and spatial learning in rats. Front Mol Neurosci. (2017) 10:269. doi: 10.3389/fnmol.2017.00269 28894415 PMC5581399

[99] Arbab T Pennartz CMA Battaglia FP. Impaired hippocampal representation of place in the Fmr1-knockout mouse model of fragile X syndrome. Sci Rep. (2018) 8:8889. doi: 10.1038/s41598-018-26853-z 29892074 PMC5995880

[100] Jeon SJ Kwon H Bae HJ Gonzales EL Kim J Chung HJ. Agmatine relieves behavioral impairments in Fragile X mice model. Neuropharmacology. (2022) 219:109234. doi: 10.1016/j.neuropharm.2022.109234 36057317

[101] Prieto M Folci A Poupon G Schiavi S Buzzelli V Pronot M. Missense mutation of Fmr1 results in impaired AMPAR-mediated plasticity and socio-cognitive deficits in mice. Nat Commun. (2021) 12:1557. doi: 10.1038/s41467-021-21820-1 33692361 PMC7946954

[102] Reinhard SM Rais M Afroz S Hanania Y Pendi K Espinoza K. Reduced perineuronal net expression in Fmr1 KO mice auditory cortex and amygdala is linked to impaired fear-associated memory. Neurobiol Learn Mem. (2019) 164:107042. doi: 10.1016/j.nlm.2019.107042 31326533 PMC7519848

[103] Sawicka K Hale CR Park CY Fak JJ Gresack JE Van Driesche SJ. FMRP has a cell-type-specific role in CA1 pyramidal neurons to regulate autism-related transcripts and circadian memory. Elife. (2019) 8:e46919. doi: 10.7554/eLife.46919 31860442 PMC6924960

[104] Boone CE Davoudi H Harrold JB Foster DJ. Abnormal sleep architecture and hippocampal circuit dysfunction in a mouse model of fragile X syndrome. Neuroscience. (2018) 384:275–89. doi: 10.1016/j.neuroscience.2018.05.012 29775702

[105] Uutela M Lindholm J Rantamäki T Umemori J Hunter K Võikar V. Distinctive behavioral and cellular responses to fluoxetine in the mouse model for Fragile X syndrome. Front Cell Neurosci. (2014) 8:150. doi: 10.3389/fncel.2014.00150 24904293 PMC4036306

[106] Qin M Entezam A Usdin K Huang T Liu ZH Hoffman GE. A mouse model of the fragile X premutation: effects on behavior, dendrite morphology, and regional rates of cerebral protein synthesis. Neurobiol Dis. (2011) 42:85–98. doi: 10.1016/j.nbd.2011.01.008 21220020 PMC3150744

[107] Hao S Tang B Wu Z Ure K Sun Y Tao H. Forniceal deep brain stimulation rescues hippocampal memory in Rett syndrome mice. Nature. (2015) 526:430–34. doi: 10.1038/nature15694 PMC4828032 26469053

[108] Moretti P Levenson JM Battaglia F Atkinson R Teague R Antalffy B. Learning and memory and synaptic plasticity are impaired in a mouse model of Rett syndrome. J Neurosci. (2006) 26:319–27. doi: 10.1523/JNEUROSCI.2623-05.2006 PMC6674314 16399702

[109] Bertoldi ML Zalosnik MI Fabio MC Aja S Roth GA Ronnett GV. MeCP2 deficiency disrupts kainate-induced presynaptic plasticity in the mossy fiber projections in the hippocampus. Front Cell Neurosci. (2019) 13:286. doi: 10.3389/fncel.2019.00286 31333414 PMC6619486

[110] Li W Bellot-Saez A Phillips ML Yang T Longo FM Pozzo-Miller L. A small-molecule TrkB ligand restores hippocampal synaptic plasticity and object location memory in Rett syndrome mice. Dis Model Mech. (2017) 10:837–45. doi: 10.1242/dmm.029959 PMC5536912 28679669

[111] He L Caudill MS Jing J Wang W Sun Y Tang J. A weakened recurrent circuit in the hippocampus of Rett syndrome mice disrupts long-term memory representations. Neuron. (2022) 110:1689–1699.e6. doi: 10.1016/j.neuron.2022.02.014 35290792 PMC9930308

[112] Kee SE Mou X Zoghbi HY Ji D. Impaired spatial memory codes in a mouse model of Rett syndrome. Elife. (2018) 7:e31451. doi: 10.7554/eLife.31451 30028675 PMC6054527

[113] Calfa G Li W Rutherford JM Pozzo-Miller L. Excitation/inhibition imbalance and impaired synaptic inhibition in hippocampal area CA3 of Mecp2 knockout mice. Hippocampus. (2015) 25:159–68. doi: 10.1002/hipo.22360 PMC4300269 25209930

[114] Sun Y Gao Y Tidei JJ Shen M Hoang JT Wagner DF. Loss of MeCP2 in immature neurons leads to impaired network integration. Hum Mol Genet. (2019) 28:245–57. doi: 10.1093/hmg/ddy338 PMC6322069 30277526

[115] Balakrishnan S Mironov SL. Regenerative glutamate release in the hippocampus of Rett syndrome model mice. PloS One. (2018) 13:e0202802. doi: 10.1371/journal.pone.0202802 30256804 PMC6157837

[116] Li W Xu X Pozzo-Miller L. Excitatory synapses are stronger in the hippocampus of Rett syndrome mice due to altered synaptic trafficking of AMPA-type glutamate receptors. Proc Natl Acad Sci USA. (2016) 113:E1575–84. doi: 10.1073/pnas.1517244113 PMC4801299 26929363

[117] Nelson ED Kavalali ET Monteggia LM. MeCP2-dependent transcriptional repression regulates excitatory neurotransmission. Curr Biol. (2006) 16:710–6. doi: 10.1016/j.cub.2006.02.062 16581518

[118] Lu H Ash RT He L Kee SE Wang W Yu D. Loss and gain of meCP2 cause similar hippocampal circuit dysfunction that is rescued by deep brain stimulation in a rett syndrome mouse model. Neuron. (2016) 91:739–47. doi: 10.1016/j.neuron.2016.07.018 PMC5019177 27499081

[119] Zhang L He J Jugloff DG Eubanks JH. The MeCP2-null mouse hippocampus displays altered basal inhibitory rhythms and is prone to hyperexcitability. Hippocampus. (2008) 18:294–309. doi: 10.1002/hipo.20389 18058824

[120] Chapleau CA Boggio EM Calfa G Percy AK Giustetto M Pozzo-Miller L. Hippocampal CA1 pyramidal neurons of Mecp2 mutant mice show a dendritic spine phenotype only in the presymptomatic stage. Neural Plast. (2012) 2012:976164. doi: 10.1155/2012/976164 22919518 PMC3418521

[121] Kaphzan H Buffington SA Ramaraj AB Lingrel JB Rasband MN Santini E. Genetic reduction of the α1 subunit of Na/K-ATPase corrects multiple hippocampal phenotypes in Angelman syndrome. Cell Rep. (2013) 4:405–12. doi: 10.1016/j.celrep.2013.07.005 PMC3756897 23911285

[122] Rayi PR Koyavski L Chakraborty D Bagrov A Kaphzan H. α1-Na/K-ATPase inhibition rescues aberrant dendritic calcium dynamics and memory deficits in the hippocampus of an Angelman syndrome mouse model. Prog Neurobiol. (2019) 182:101676. doi: 10.1016/j.pneurobio.2019.101676 31401139

[123] van Woerden GM Harris KD Hojjati MR Gustin RM Qiu S de Avila Freire R. Rescue of neurological deficits in a mouse model for Angelman syndrome by reduction of alphaCaMKII inhibitory phosphorylation. Nat Neurosci. (2007) 10:280–2. doi: 10.1038/nn1845 17259980

[124] Cao C Rioult-Pedotti MS Migani P Yu CJ Tiwari R Parang K. Impairment of TrkB-PSD-95 signaling in Angelman syndrome. PloS Biol. (2013) 11:e1001478. doi: 10.1371/journal.pbio.1001478 23424281 PMC3570550

[125] Kaphzan H Buffington SA Jung JI Rasband MN Klann E. Alterations in intrinsic membrane properties and the axon initial segment in a mouse model of Angelman syndrome. J Neurosci. (2011) 31:17637–48. doi: 10.1523/JNEUROSCI.4162-11.2011 PMC3483031 22131424

[126] Sun J Zhu G Liu Y Standley S Ji A Tunuguntla R. UBE3A regulates synaptic plasticity and learning and memory by controlling SK2 channel endocytosis. Cell Rep. (2015) 12:449–61. doi: 10.1016/j.celrep.2015.06.023 PMC4520703 26166566

[127] Viho EMG Punt AM Distel B Houtman R Kroon J Elgersma Y. The hippocampal response to acute corticosterone elevation is altered in a mouse model for angelman syndrome. Int J Mol Sci. (2022) 24:303. doi: 10.3390/ijms24010303 36613751 PMC9820460

[128] Godavarthi SK Dey P Maheshwari M Jana NR. Defective glucocorticoid hormone receptor signaling leads to increased stress and anxiety in a mouse model of Angelman syndrome. Hum Mol Genet. (2012) 21:1824–34. doi: 10.1093/hmg/ddr614 22215440

[129] Godavarthi SK Sharma A Jana NR. Reversal of reduced parvalbumin neurons in hippocampus and amygdala of Angelman syndrome model mice by chronic treatment of fluoxetine. J Neurochem. (2014) 130:444–54. doi: 10.1111/jnc.12726 24678582

[130] Curatolo P Scheper M Emberti Gialloreti L Specchio N Aronica E. Is tuberous sclerosis complex-associated autism a preventable and treatable disorder? World J Pediatr. (2024) 20:40–53. doi: 10.1007/s12519-023-00762-2 37878130

[131] Haji N Riebe I Aguilar-Valles A Artinian J Laplante I Lacaille JC. Tsc1 haploinsufficiency in Nkx2.1 cells upregulates hippocampal interneuron mTORC1 activity, impairs pyramidal cell synaptic inhibition, and alters contextual fear discrimination and spatial working memory in mice. Mol Autism. (2020) 11:29. doi: 10.1186/s13229-020-00340-7 32375878 PMC7201610

[132] Ehninger D Han S Shilyansky C Zhou Y Li W Kwiatkowski DJ. Reversal of learning deficits in a Tsc2+/- mouse model of tuberous sclerosis. Nat Med. (2008) 14:843–8. doi: 10.1038/nm1788 PMC2664098 18568033

[133] Goorden SM van Woerden GM van der Weerd L Cheadle JP Elgersma Y. Cognitive deficits in Tsc1+/- mice in the absence of cerebral lesions and seizures. Ann Neurol. (2007) 62:648–55. doi: 10.1002/ana.21317 18067135

[134] Bateup HS Johnson CA Denefrio CL Saulnier JL Kornacker K Sabatini BL. Excitatory/inhibitory synaptic imbalance leads to hippocampal hyperexcitability in mouse models of tuberous sclerosis. Neuron. (2013) 78:510–22. doi: 10.1016/j.neuron.2013.03.017 PMC3690324 23664616

[135] Koene LM Niggl E Wallaard I Proietti-Onori M Rotaru DC Elgersma Y. Identifying the temporal electrophysiological and molecular changes that contribute to TSC-associated epileptogenesis. JCI Insight. (2021) 6:e150120. doi: 10.1172/jci.insight.150120 34877936 PMC8675202

[136] Lasarge CL Danzer SC. Mechanisms regulating neuronal excitability and seizure development following mTOR pathway hyperactivation. Front Mol Neurosci. (2014) 7:18. doi: 10.3389/fnmol.2014.00018 24672426 PMC3953715

[137] Wong M Ess KC Uhlmann EJ Jansen LA Li W Crino PB. Impaired glial glutamate transport in a mouse tuberous sclerosis epilepsy model. Ann Neurol. (2003) 54:251–6. doi: 10.1002/ana.10648 12891680

[138] Xu L Zeng LH Wong M. Impaired astrocytic gap junction coupling and potassium buffering in a mouse model of tuberous sclerosis complex. Neurobiol Dis. (2009) 34:291–9. doi: 10.1016/j.nbd.2009.01.010 PMC2764295 19385061

[139] Zhang B Zou J Han L Rensing N Wong M. Microglial activation during epileptogenesis in a mouse model of tuberous sclerosis complex. Epilepsia. (2016) 57:1317–25. doi: 10.1111/epi.13429 PMC4972670 27263494

[140] Deng Y Yang Q Yang Y Li Y Peng H Wu S. Conditional knockout of Tsc1 in RORγt-expressing cells induces brain damage and early death in mice. J Neuroinflamm. (2021) 18:107. doi: 10.1186/s12974-021-02153-8 PMC8101034 33957945

[141] Chévere-Torres I Kaphzan H Bhattacharya A Kang A Maki JM Gambello MJ. Metabotropic glutamate receptor-dependent long-term depression is impaired due to elevated ERK signaling in the ΔRG mouse model of tuberous sclerosis complex. Neurobiol Dis. (2012) 45:1101–10. doi: 10.1016/j.nbd.2011.12.028 PMC3276695 22198573

[142] Potter WB Basu T O'Riordan KJ Kirchner A Rutecki P Burger C. Reduced juvenile long-term depression in tuberous sclerosis complex is mitigated in adults by compensatory recruitment of mGluR5 and Erk signaling. PloS Biol. (2013) 11:e1001627. doi: 10.1371/journal.pbio.1001627 23966835 PMC3742461

[143] Bateup HS Takasaki KT Saulnier JL Denefrio CL Sabatini BL. Loss of Tsc1 in *vivo* impairs hippocampal mGluR-LTD and increases excitatory synaptic function. J Neurosci. (2011) 31:8862–9. doi: 10.1523/JNEUROSCI.1617-11.2011 PMC3133739 21677170

[144] Tavazoie SF Alvarez VA Ridenour DA Kwiatkowski DJ Sabatini BL. Regulation of neuronal morphology and function by the tumor suppressors Tsc1 and Tsc2. Nat Neurosci. (2005) 8:1727–34. doi: 10.1038/nn1566 16286931

[145] Weiss HR Liu X Chi OZ. Cerebral O(2) consumption in young Eker rats, effects of GABA blockade: implications for autism. Int J Dev Neurosci. (2008) 26:517–21. doi: 10.1016/j.ijdevneu.2008.01.002 18282678

[146] Chi OZ Wu CC Liu X Rah KH Jacinto E Weiss HR. Restoration of normal cerebral oxygen consumption with rapamycin treatment in a rat model of autism-tuberous sclerosis. Neuromol Med. (2015) 17:305–13. doi: 10.1007/s12017-015-8359-5 PMC4888058 26048361

[147] Harony-Nicolas H Kay M du Hoffmann J Klein ME Bozdagi-Gunal O Riad M. Oxytocin improves behavioral and electrophysiological deficits in a novel Shank3-deficient rat. Elife. (2017) 6:e18904. doi: 10.7554/eLife.18904 28139198 PMC5283828

[148] Atanasova E Arévalo AP Graf I Zhang R Bockmann J Lutz AK. Immune activation during pregnancy exacerbates ASD-related alterations in Shank3-deficient mice. Mol Autism. (2023) 14:1. doi: 10.1186/s13229-022-00532-3 36604742 PMC9814193

[149] Wang X McCoy PA Rodriguiz RM Pan Y Je HS Roberts AC. Synaptic dysfunction and abnormal behaviors in mice lacking major isoforms of Shank3. Hum Mol Genet. (2011) 20:3093–108. doi: 10.1093/hmg/ddr212 PMC3131048 21558424

[150] Oberman LM Boccuto L Cascio L Sarasua S Kaufmann WE. Autism spectrum disorder in Phelan-McDermid syndrome: initial characterization and genotype-phenotype correlations. Orphanet J Rare Dis. (2015) 10:105. doi: 10.1186/s13023-015-0323-9 26306707 PMC4549933

[151] Urrutia-Ruiz C Rombach D Cursano S Gerlach-Arbeiter S Schoen M Bockmann J. Deletion of the autism-associated protein SHANK3 abolishes structural synaptic plasticity after brain trauma. Int J Mol Sci. (2022) 23:6081. doi: 10.3390/ijms23116081 35682760 PMC9181590

[152] Xu D Meng Y An S Meng W Li H Zhang W. Swimming exercise is a promising early intervention for autism-like behavior in Shank3 deletion rats. CNS Neurosci Ther. (2023) 29:78–90. doi: 10.1111/cns.13920 36221783 PMC9804047

[153] Lee J Chung C Ha S Lee D Kim DY Kim H. Shank3-mutant mice lacking exon 9 show altered excitation/inhibition balance, enhanced rearing, and spatial memory deficit. Front Cell Neurosci. (2015) 9:94. doi: 10.3389/fncel.2015.00094 25852484 PMC4365696

[154] Yoo YE Yoo T Lee S Lee J Kim D Han HM. Shank3 mice carrying the human Q321R mutation display enhanced self-grooming, abnormal electroencephalogram patterns, and suppressed neuronal excitability and seizure susceptibility. Front Mol Neurosci. (2019) 12:155. doi: 10.3389/fnmol.2019.00155 31275112 PMC6591539

[155] Jaramillo TC Speed HE Xuan Z Reimers JM Liu S Powell CM. Altered striatal synaptic function and abnormal behaviour in shank3 exon4-9 deletion mouse model of autism. Autism Res. (2016) 9:350–75. doi: 10.1002/aur.1529 PMC4857590 26559786

[156] Jaramillo TC Speed HE Xuan Z Reimers JM Escamilla CO Weaver TP. Novel Shank3 mutant exhibits behaviors with face validity for autism and altered striatal and hippocampal function. Autism Res. (2017) 10:42–65. doi: 10.1002/aur.1664 27492494 PMC5274551

[157] Bozdagi O Sakurai T Papapetrou D Wang X Dickstein DL Takahashi N. Haploinsufficiency of the autism-associated Shank3 gene leads to deficits in synaptic function, social interaction, and social communication. Mol Autism. (2010) 1:15. doi: 10.1186/2040-2392-1-15 21167025 PMC3019144

[158] Tao K Chung M Watarai A Huang Z Wang MY Okuyama T. Disrupted social memory ensembles in the ventral hippocampus underlie social amnesia in autism-associated Shank3 mutant mice. Mol Psychiatry. (2022) 27:2095–105. doi: 10.1038/s41380-021-01430-5 PMC9126818 35115700

[159] Peça J Feliciano C Ting JT Wang W Wells MF Venkatraman TN. Shank3 mutant mice display autistic-like behaviours and striatal dysfunction. Nature. (2011) 472:437–42. doi: 10.1038/nature09965 PMC3090611 21423165

[160] Cope EC Wang SH Waters RC Gore IR Vasquez B Laham BJ. Activation of the CA2-ventral CA1 pathway reverses social discrimination dysfunction in Shank3B knockout mice. Nat Commun. (2023) 14:1750. doi: 10.1038/s41467-023-37248-8 36991001 PMC10060401

[161] Yoo T Yoo YE Kang H Kim E. Age, brain region, and gene dosage-differential transcriptomic changes in Shank3-mutant mice. Front Mol Neurosci. (2022) 15:1017512. doi: 10.3389/fnmol.2022.1017512 36311023 PMC9597470

[162] Dahlhaus R Hines RM Eadie BD Kannangara TS Hines DJ Brown CE. Overexpression of the cell adhesion protein neuroligin-1 induces learning deficits and impairs synaptic plasticity by altering the ratio of excitation to inhibition in the hippocampus. Hippocampus. (2010) 20:305–22. doi: 10.1002/hipo.20630 19437420

[163] Jedlicka P Vnencak M Krueger DD Jungenitz T Brose N Schwarzacher SW. Neuroligin-1 regulates excitatory synaptic transmission, LTP and EPSP-spike coupling in the dentate gyrus. vivo. Brain Struct Funct. (2015) 220:47–58. doi: 10.1007/s00429-013-0636-1 25713840

[164] Jiang M Polepalli J Chen LY Zhang B Südhof TC Malenka RC. Conditional ablation of neuroligin-1 in CA1 pyramidal neurons blocks LTP by a cell-autonomous NMDA receptor-independent mechanism. Mol Psychiatry. (2017) 22:375–83. doi: 10.1038/mp.2016.80 PMC5122464 27217145

[165] Chubykin AA Atasoy D Etherton MR Brose N Kavalali ET Gibson JR. Activity-dependent validation of excitatory versus inhibitory synapses by neuroligin-1 versus neuroligin-2. Neuron. (2007) 54:919–31. doi: 10.1016/j.neuron.2007.05.029 PMC3738748 17582332

[166] Jedlicka P Hoon M Papadopoulos T Vlachos A Winkels R Poulopoulos A. Increased dentate gyrus excitability in neuroligin-2-deficient mice. vivo. Cereb Cortex. (2011) 21:357–67. doi: 10.1093/cercor/bhq100 20530218

[167] Kohl C Riccio O Grosse J Zanoletti O Fournier C Schmidt MV. Hippocampal neuroligin-2 overexpression leads to reduced aggression and inhibited novelty reactivity in rats. PloS One. (2013) 8:e56871. doi: 10.1371/journal.pone.0056871 23451101 PMC3579928

[168] Van Zandt M Weiss E Almyasheva A Lipior S Maisel S Naegele JR. Adeno-associated viral overexpression of neuroligin 2 in the mouse hippocampus enhances GABAergic synapses and impairs hippocampus-dependent behaviors. Behav Brain Res. (2019) 362:7–20. doi: 10.1016/j.bbr.2018.12.052 30605713 PMC6626304

[169] Koganezawa N Hanamura K Schwark M Krueger-Burg D Kawabe H. Super-resolved 3D-STED microscopy identifies a layer-specific increase in excitatory synapses in the hippocampal CA1 region of Neuroligin-3 KO mice. Biochem Biophys Res Commun. (2021) 582:144–9. doi: 10.1016/j.bbrc.2021.10.003 34715405

[170] Modi B Pimpinella D Pazienti A Zacchi P Cherubini E Griguoli M. Possible implication of the CA2 hippocampal circuit in social cognition deficits observed in the neuroligin 3 knock-out mouse, a non-syndromic animal model of autism. Front Psychiatry. (2019) 10:513. doi: 10.3389/fpsyt.2019.00513 31379628 PMC6659102

[171] Polepalli JS Wu H Goswami D Halpern CH Südhof TC Malenka RC. Modulation of excitation on parvalbumin interneurons by neuroligin-3 regulates the hippocampal network. Nat Neurosci. (2017) 20:219–29. doi: 10.1038/nn.4471 PMC5272845 28067903

[172] Etherton M Földy C Sharma M Tabuchi K Liu X Shamloo M. Autism-linked neuroligin-3 R451C mutation differentially alters hippocampal and cortical synaptic function. Proc Natl Acad Sci USA. (2011) 108:13764–9. doi: 10.1073/pnas.1111093108 PMC3158170 21808020

[173] Tabuchi K Chang W Asgar NF Pramanik G. Synapse maturation and autism: learning from neuroligin model mice. Nihon Shinkei Seishin Yakurigaku Zasshi. (2014) 34:1–4. 25069265

[174] Tabuchi K Blundell J Etherton MR Hammer RE Liu X Powell CM. A neuroligin-3 mutation implicated in autism increases inhibitory synaptic transmission in mice. Science. (2007) 318:71–6. doi: 10.1126/science.1146221 PMC3235367 17823315

[175] Sgritta M Vignoli B Pimpinella D Griguoli M Santi S Bialowas A. Impaired synaptic plasticity in an animal model of autism exhibiting early hippocampal GABAergic-BDNF/TrkB signaling alterations. iScience. (2022) 26:105728. doi: 10.1016/j.isci.2022.105728 36582822 PMC9793278

[176] Hammer M Krueger-Burg D Tuffy LP Cooper BH Taschenberger H Goswami SP. Perturbed hippocampal synaptic inhibition and γ-oscillations in a neuroligin-4 knockout mouse model of autism. Cell Rep. (2015) 13:516–23. doi: 10.1016/j.celrep.2015.09.011 PMC5862414 26456829

[177] Muellerleile J Vnencak M Sethi MVA Jungenitz T Schwarzacher SW Jedlicka P. Increased network inhibition in the dentate gyrus of adult neuroligin-4 knock-out mice. eNeuro. (2023) 10:ENEURO.0471–22.2023. doi: 10.1523/ENEURO.0471-22.2023 PMC10121080 37080762

[178] Guneykaya D Ugursu B Logiacco F Popp O Feiks MA Meyer N. Sex-specific microglia state in the Neuroligin-4 knock-out mouse model of autism spectrum disorder. Brain Behav Immun. (2023) 111:61–75. doi: 10.1016/j.bbi.2023.03.023 37001827 PMC10330133

[179] Yonan JM Steward O. Vector-mediated PTEN deletion in the adult dentate gyrus initiates new growth of granule cell bodies and dendrites and expansion of mossy fiber terminal fields that continues for months. Neurobiol Dis. (2023) 184:106190. doi: 10.1016/j.nbd.2023.106190 37290578

[180] Amiri A Cho W Zhou J Birnbaum SG Sinton CM McKay RM. Pten deletion in adult hippocampal neural stem/progenitor cells causes cellular abnormalities and alters neurogenesis. J Neurosci. (2012) 32:5880–90. doi: 10.1523/JNEUROSCI.5462-11.2012 PMC6703621 22539849

[181] Luikart BW Schnell E Washburn EK Bensen AL Tovar KR Westbrook GL. Pten knockdown in *vivo* increases excitatory drive onto dentate granule cells. J Neurosci. (2011) 31:4345–54. doi: 10.1523/JNEUROSCI.0061-11.2011 PMC3113533 21411674

[182] Matsushita Y Sakai Y Shimmura M Shigeto H Nishio M Akamine S. Hyperactive mTOR signals in the proopiomelanocortin-expressing hippocampal neurons cause age-dependent epilepsy and premature death in mice. Sci Rep. (2016) 6:22991. doi: 10.1038/srep22991 26961412 PMC4785342

[183] LaSarge CL Santos VR Danzer SC. PTEN deletion from adult-generated dentate granule cells disrupts granule cell mossy fiber axon structure. Neurobiol Dis. (2015) 75:142–50. doi: 10.1016/j.nbd.2014.12.029 PMC4351143 25600212

[184] Santos VR Pun RYK Arafa SR LaSarge CL Rowley S Khademi S. PTEN deletion increases hippocampal granule cell excitability in male and female mice. Neurobiol Dis. (2017) 108:339–51. doi: 10.1016/j.nbd.2017.08.014 PMC5675774 28855130

[185] Takeuchi K Gertner MJ Zhou J Parada LF Bennett MV Zukin RS. Dysregulation of synaptic plasticity precedes appearance of morphological defects in a Pten conditional knockout mouse model of autism. Proc Natl Acad Sci USA. (2013) 110:4738–43. doi: 10.1073/pnas.1222803110 PMC3607034 23487788

[186] LaSarge CL Pun RY Muntifering MB Danzer SC. Disrupted hippocampal network physiology following PTEN deletion from newborn dentate granule cells. Neurobiol Dis. (2016) 96:105–14. doi: 10.1016/j.nbd.2016.09.004 PMC5102799 27597527

[187] Sperow M Berry RB Bayazitov IT Zhu G Baker SJ Zakharenko SS. Phosphatase and tensin homologue. (PTEN) regulates synaptic plasticity independently of its effect on neuronal morphology and migration. J Physiol. (2012) 590:777–92. doi: 10.1113/jphysiol.2011.220236 PMC3381310 22147265

[188] Kind PC Bird A. CDKL5 deficiency disorder: a pathophysiology of neural maintenance. J Clin Invest. (2021) 131:e153606. doi: 10.1172/JCI153606 34720088 PMC8553564

[189] Fuchs C Rimondini R Viggiano R Trazzi S De Franceschi M Bartesaghi R. Inhibition of GSK3β rescues hippocampal development and learning in a mouse model of CDKL5 disorder. Neurobiol Dis. (2015) 82:298–310. doi: 10.1016/j.nbd.2015.06.018 26143616

[190] Fuchs C Trazzi S Torricella R Viggiano R De Franceschi M Amendola E. Loss of CDKL5 impairs survival and dendritic growth of newborn neurons by altering AKT/GSK-3β signaling. Neurobiol Dis. (2014) 70:53–68. doi: 10.1016/j.nbd.2014.06.006 24952363 PMC4146476

[191] Gennaccaro L Fuchs C Loi M Pizzo R Alvente S Berteotti C. Age-related cognitive and motor decline in a mouse model of CDKL5 deficiency disorder is associated with increased neuronal senescence and death. Aging Dis. (2021) 12:764–85. doi: 10.14336/AD.2020.0827 PMC8139207 34094641

[192] Galvani G Mottolese N Gennaccaro L Loi M Medici G Tassinari M. Inhibition of microglia overactivation restores neuronal survival in a mouse model of CDKL5 deficiency disorder. J Neuroinflamm. (2021) 18:155. doi: 10.1186/s12974-021-02204-0 PMC8265075 34238328

[193] Fuchs C Medici G Trazzi S Gennaccaro L Galvani G Berteotti C. CDKL5 deficiency predisposes neurons to cell death through the deregulation of SMAD3 signaling. Brain Pathol. (2019) 29:658–74. doi: 10.1111/bpa.12716 PMC8028508 30793413

[194] Zhu ZA Li YY Xu J Xue H Feng X Zhu YC. CDKL5 deficiency in adult glutamatergic neurons alters synaptic activity and causes spontaneous seizures *via* TrkB signaling. Cell Rep. (2023) 42:113202. doi: 10.1016/j.celrep.2023.113202 37777961

[195] Okuda K Kobayashi S Fukaya M Watanabe A Murakami T Hagiwara M. CDKL5 controls postsynaptic localization of GluN2B-containing NMDA receptors in the hippocampus and regulates seizure susceptibility. Neurobiol Dis. (2017) 106:158–70. doi: 10.1016/j.nbd.2017.07.002 28688852

[196] Nicolini C Fahnestock M. The valproic acid-induced rodent model of autism. Exp Neurol. (2018) 299:217–27. doi: 10.1016/j.expneurol.2017.04.017 28472621

[197] Gouda B Sinha SN Chalamaiah M Vakdevi V Shashikala P Veeresh B. Sex differences in animal models of sodium-valproate-induced autism in postnatal BALB/c mice: whole-brain histoarchitecture and 5-HT2A receptor biomarker evidence. Biology. (2022) 11:79. doi: 10.3390/biology11010079 35053076 PMC8772829

[198] Matsuo K Yabuki Y Fukunaga K. 5-aminolevulinic acid inhibits oxidative stress and ameliorates autistic-like behaviors in prenatal valproic acid-exposed rats. Neuropharmacology. (2020) 168:107975. doi: 10.1016/j.neuropharm.2020.107975 31991146

[199] Sandhu A Rawat K Gautam V Sharma A Kumar A Saha L. Phosphodiesterase inhibitor, ibudilast alleviates core behavioral and biochemical deficits in the prenatal valproic acid exposure model of autism spectrum disorder. Brain Res. (2023) 1815:148443. doi: 10.1016/j.brainres.2023.148443 37290608

[200] Singla R Mishra A Joshi R Kumar R Sarma P Sharma AR. Inhibition of the ERK1/2 phosphorylation by dextromethorphan protects against core autistic symptoms in VPA induced autistic rats: in silico and in vivo drug repurposition study. ACS Chem Neurosci. (2021) 12:1749–67. doi: 10.1021/acschemneuro.0c00672 33913688

[201] Zohny SM Habib MZ Mohamad MI Elayat WM Elhossiny RM El-Salam MFA. Memantine/aripiprazole combination alleviates cognitive dysfunction in valproic acid rat model of autism: hippocampal CREB/BDNF signaling and glutamate homeostasis. Neurotherapeutics. (2023) 20:464–83. doi: 10.1007/s13311-023-01360-w PMC10121975 36918475

[202] Taheri F Esmaeilpour K Sepehri G Sheibani V Ur Rehman N Maneshian M. Histamine H3 receptor antagonist, ciproxifan, alleviates cognition and synaptic plasticity alterations in a valproic acid-induced animal model of autism. Psychopharmacology. (2022) 239:2673–93. doi: 10.1007/s00213-022-06155-z 35538250

[203] Lima-Castañeda LÁ Bringas ME Aguilar-Hernandez L Garcés-Ramírez L Morales-Medina JC Flores G. The antipsychotic olanzapine reduces memory deficits and neuronal abnormalities in a male rat model of Autism. J Chem Neuroanat. (2023) 132:102317. doi: 10.1016/j.jchemneu.2023.102317 37482145

[204] Seo TB Cho HS Shin MS Kim CJ Ji ES Baek SS. Treadmill exercise improves behavioral outcomes and spatial learning memory through up-regulation of reelin signaling pathway in autistic rats. J Exerc Rehabil. (2013) 9:220–9. doi: 10.12965/jer.130003 PMC3836510 24278864

[205] Zhang Y Xiang Z Jia Y He X Wang L Cui W. The Notch signaling pathway inhibitor Dapt alleviates autism-like behavior, autophagy and dendritic spine density abnormalities in a valproic acid-induced animal model of autism. Prog Neuropsychopharmacol Biol Psychiatry. (2019) 94:109644. doi: 10.1016/j.pnpbp.2019.109644 31075347

[206] Hou Q Wang Y Li Y Chen D Yang F Wang S. A developmental study of abnormal behaviors and altered GABAergic signaling in the VPA-treated rat model of autism. Front Behav Neurosci. (2018) 12:182. doi: 10.3389/fnbeh.2018.00182 30186123 PMC6110947

[207] Hara Y Ago Y Taruta A Hasebe S Kawase H Tanabe W. Risperidone and aripiprazole alleviate prenatal valproic acid-induced abnormalities in behaviors and dendritic spine density in mice. Psychopharmacology. (2017) 234:3217–28. doi: 10.1007/s00213-017-4703-9 28798977

[208] Barzegari A Amouzad Mahdirejei H Hanani M Esmaeili MH Salari AA. Adolescent swimming exercise following maternal valproic acid treatment improves cognition and reduces stress-related symptoms in offspring mice: Role of sex and brain cytokines. Physiol Behav. (2023) 269:114264. doi: 10.1016/j.physbeh.2023.114264 37295664

[209] Yamaguchi H Hara Y Ago Y Takano E Hasebe S Nakazawa T. Environmental enrichment attenuates behavioral abnormalities in valproic acid-exposed autism model mice. Behav Brain Res. (2017) 333:67–73. doi: 10.1016/j.bbr.2017.06.035 28655565

[210] de Oliveira Ferreira E Pessoa Gomes JM Neves KRT Lima FAV de Barros Viana GS de Andrade GM. Maternal treatment with aripiprazole prevents the development of a valproic acid-induced autism-like phenotype in juvenile male mice. Behav Pharmacol. (2023) 34:154–68. doi: 10.1097/FBP.0000000000000718 36853856

[211] Wang J Xu C Liu C Zhou Q Chao G Jin Y. Effects of different doses of lithium on the central nervous system in the rat valproic acid model of autism. Chem Biol Interact. (2023) 370:110314. doi: 10.1016/j.cbi.2022.110314 36535311

[212] Gąssowska-Dobrowolska M Cieślik M Czapski GA Jęśko H Frontczak-Baniewicz M Gewartowska M. Prenatal exposure to valproic acid affects microglia and synaptic ultrastructure in a brain-region-specific manner in young-adult male rats: relevance to autism spectrum disorders. Int J Mol Sci. (2020) 21:3576. doi: 10.3390/ijms21103576 32443651 PMC7279050

[213] Luhach K Kulkarni GT Singh VP Sharma B. Vinpocetine amended prenatal valproic acid induced features of ASD possibly by altering markers of neuronal function, inflammation, and oxidative stress. Autism Res. (2021) 14:2270–86. doi: 10.1002/aur.2597 34415116

[214] Luhach K Kulkarni GT Singh VP Sharma B. Cilostazol attenuated prenatal valproic acid-induced behavioural and biochemical deficits in a rat model of autism spectrum disorder. J Pharm Pharmacol. (2021) 73:1460–9. doi: 10.1093/jpp/rgab115 34459916

[215] Luhach K Kulkarni GT Singh VP Sharma B. Attenuation of neurobehavioural abnormalities by papaverine in prenatal valproic acid rat model of ASD. Eur J Pharmacol. (2021) 890:173663. doi: 10.1016/j.ejphar.2020.173663 33127361

[216] Eissa N Azimullah S Jayaprakash P Jayaraj RL Reiner D Ojha SK. The dual-active histamine H3 receptor antagonist and acetylcholine esterase inhibitor E100 ameliorates stereotyped repetitive behavior and neuroinflammmation in sodium valproate induced autism in mice. Chem Biol Interact. (2019) 312:108775. doi: 10.1016/j.cbi.2019.108775 31369746

[217] Gifford JJ Deshpande P Mehta P Wagner GC Kusnecov AW. The effect of valproic acid exposure throughout development on microglia number in the prefrontal cortex, hippocampus and cerebellum. Neuroscience. (2022) 481:166–77. doi: 10.1016/j.neuroscience.2021.11.012 34780921

[218] Bronzuoli MR Facchinetti R Ingrassia D Sarvadio M Schiavi S Steardo L. Neuroglia in the autistic brain: evidence from a preclinical model. Mol Autism. (2018) 9:66. doi: 10.1186/s13229-018-0254-0 30603062 PMC6307226

[219] Habib MZ Elnahas EM Aboul-Ela YM Ebeid MA Tarek M Sadek DR. Risperidone impedes glutamate excitotoxicity in a valproic acid rat model of autism: Role of ADAR2 in AMPA GluA2 RNA editing. Eur J Pharmacol. (2023) 955:175916. doi: 10.1016/j.ejphar.2023.175916 37460052

[220] Zhang J Zhang JX Zhang QL. PI3K/AKT/mTOR-mediated autophagy in the development of autism spectrum disorder. Brain Res Bull. (2016) 125:152–8. doi: 10.1016/j.brainresbull.2016.06.007 27320472

[221] Zhang J Liu LM Ni JF. Rapamycin modulated brain-derived neurotrophic factor and B-cell lymphoma 2 to mitigate autism spectrum disorder in rats. Neuropsychiatr Dis Treat. (2017) 13:835–42. doi: 10.2147/NDT.S125088 PMC5365326 28360521

[222] Choi M Ko SY Seo JY Kim DG Lee H Chung H. Autistic-like social deficits in hippocampal MeCP2 knockdown rat models are rescued by ketamine. BMB Rep. (2022) 55:238–43. doi: 10.5483/BMBRep.2022.55.5.038 PMC9152577 35410641

[223] Yu X Mostafijur Rahman M Carter SA Lin JC Zhuang Z Chow T. Prenatal air pollution, maternal immune activation, and autism spectrum disorder. Environ Int. (2023) 179:108148. doi: 10.1016/j.envint.2023.108148 37595536 PMC10792527

[224] Ardalan M Chumak T Quist A Hermans E Hoseinpoor Rafati A Gravina G. Reelin cells and sex-dependent synaptopathology in autism following postnatal immune activation. Br J Pharmacol. (2022) 179:4400–22. doi: 10.1111/bph.15859 PMC9545289 35474185

[225] Fernández de Cossío L Guzmán A van der Veldt S Luheshi GN. Prenatal infection leads to ASD-like behavior and altered synaptic pruning in the mouse offspring. Brain Behav Immun. (2017) 63:88–98. doi: 10.1016/j.bbi.2016.09.028 27697456

[226] Ardalan M Chumak T Quist A Jabbari Shiadeh SM Mallard AJ Rafati AH. Sex-dependent gliovascular interface abnormality in the hippocampus following postnatal immune activation in mice. Dev Neurosci. (2022) 44:320–30. doi: 10.1159/000525478 PMC9533445 35705008

[227] Solmaz V Erdoğan MA Alnak A Meral A Erbaş O. Erythropoietin shows gender dependent positive effects on social deficits, learning/memory impairments, neuronal loss and neuroinflammation in the lipopolysaccharide induced rat model of autism. Neuropeptides. (2020) 83:102073. doi: 10.1016/j.npep.2020.102073 32736811

[228] Pang Y Dai X Roller A Carter K Paul I Bhatt AJ. Early postnatal lipopolysaccharide exposure leads to enhanced neurogenesis and impaired communicative functions in rats. PloS One. (2016) 11:e0164403. doi: 10.1371/journal.pone.0164403 27723799 PMC5056722

[229] Andoh M Shibata K Okamoto K Onodera J Morishita K Miura Y. Exercise reverses behavioral and synaptic abnormalities after maternal inflammation. Cell Rep. (2019) 27:2817–2825.e5. doi: 10.1016/j.celrep.2019.05.015 31167129

[230] Giovanoli S Weber-Stadlbauer U Schedlowski M Meyer U Engler H. Prenatal immune activation causes hippocampal synaptic deficits in the absence of overt microglia anomalies. Brain Behav Immun. (2016) 55:25–38. doi: 10.1016/j.bbi.2015.09.015 26408796

[231] Smolders S Smolders SM Swinnen N Gärtner A Rigo JM Legendre P. Maternal immune activation evoked by polyinosinic:polycytidylic acid does not evoke microglial cell activation in the embryo. Front Cell Neurosci. (2015) 9:301. doi: 10.3389/fncel.2015.00301 26300736 PMC4525016

[232] Pineda E Shin D You SJ Auvin S Sankar R Mazarati A. Maternal immune activation promotes hippocampal kindling epileptogenesis in mice. Ann Neurol. (2013) 74:11–9. doi: 10.1002/ana.23898 PMC3775928 23907982

[233] Wolff AR Bilkey DK. Prenatal immune activation alters hippocampal place cell firing characteristics in adult animals. Brain Behav Immun. (2015) 48:232–43. doi: 10.1016/j.bbi.2015.03.012 25843370

[234] Meyer U Nyffeler M Yee BK Knuesel I Feldon J. Adult brain and behavioral pathological markers of prenatal immune challenge during early/middle and late fetal development in mice. Brain Behav Immun. (2008) 22:469–86. doi: 10.1016/j.bbi.2007.09.012 18023140

[235] Haghani A Johnson RG Woodward NC Feinberg JI Lewis K Ladd-Acosta C. Adult mouse hippocampal transcriptome changes associated with long-term behavioral and metabolic effects of gestational air pollution toxicity. Transl Psychiatry. (2020) 10:218. doi: 10.1038/s41398-020-00907-1 32636363 PMC7341755

[236] Wang T Zhang T Sun L Li W Zhang C Yu L. Gestational B-vitamin supplementation alleviates PM2.5-induced autism-like behavior and hippocampal neurodevelopmental impairment in mice offspring. Ecotoxicol Environ Saf. (2019) 185:109686. doi: 10.1016/j.ecoenv.2019.109686 31546205

[237] Klocke C Allen JL Sobolewski M Mayer-Pröschel M Blum JL Lauterstein D. Neuropathological consequences of gestational exposure to concentrated ambient fine and ultrafine particles in the mouse. Toxicol Sci. (2017) 156:492–508. doi: 10.1093/toxsci/kfx010 28087836 PMC6074840

[238] Nephew BC Nemeth A Hudda N Beamer G Mann P Petitto J. Traffic-related particulate matter affects behavior, inflammation, and neural integrity in a developmental rodent model. Environ Res. (2020) 183:109242. doi: 10.1016/j.envres.2020.109242 32097814 PMC7167358

[239] Meyza KZ Blanchard DC. The BTBR mouse model of idiopathic autism - Current view on mechanisms. Neurosci Biobehav Rev. (2017) 76:99–110. doi: 10.1016/j.neubiorev.2016.12.037 28167097 PMC5403558

[240] Zhang Q Wu H Zou M Li L Li Q Sun C. Folic acid improves abnormal behavior *via* mitigation of oxidative stress, inflammation, and ferroptosis in the BTBR T+ tf/J mouse model of autism. J Nutr Biochem. (2019) 71:98–109. doi: 10.1016/j.jnutbio.2019.05.002 31323609

[241] Wu H Zhao G Liu S Zhang Q Wang P Cao Y. Supplementation with selenium attenuates autism-like behaviors and improves oxidative stress, inflammation and related gene expression in an autism disease model. J Nutr Biochem. (2022) 107:109034. doi: 10.1016/j.jnutbio.2022.109034 35500829

[242] Seese RR Maske AR Lynch G Gall CM. Long-term memory deficits are associated with elevated synaptic ERK1/2 activation and reversed by mGluR5 antagonism in an animal model of autism. Neuropsychopharmacology. (2014) 39:1664–73. doi: 10.1038/npp.2014.13 PMC4023139 24448645

[243] Martin LA Hsu FW Herd B Gregg M Sample H Kaplan J. Executive functions in agenesis of the corpus callosum: Working memory and sustained attention in the BTBR inbred mouse strain. Brain Behav. (2021) 11:e01933. doi: 10.1002/brb3.1933 33300691 PMC7821616

[244] Reshetnikov VV Ayriyants KA Ryabushkina YA Sozonov NG Bondar NP. Sex-specific behavioral and structural alterations caused by early-life stress in C57BL/6 and BTBR mice. Behav Brain Res. (2021) 414:113489. doi: 10.1016/j.bbr.2021.113489 34303728

[245] Stephenson DT O'Neill SM Narayan S Tiwari A Arnold E Samaroo HD. Histopathologic characterization of the BTBR mouse model of autistic-like behavior reveals selective changes in neurodevelopmental proteins and adult hippocampal neurogenesis. Mol Autism. (2011) 2:7. doi: 10.1186/2040-2392-2-7 21575186 PMC3135520

[246] Guo YP Commons KG. Serotonin neuron abnormalities in the BTBR mouse model of autism. Autism Res. (2017) 10:66–77. doi: 10.1002/aur.1665 27478061 PMC5518607

[247] Eissa N Venkatachalam K Jayaprakash P Yuvaraju P Falkenstein M Stark H. Experimental studies indicate that ST-2223, the antagonist of histamine H3 and dopamine D2/D3 receptors, restores social deficits and neurotransmission dysregulation in mouse model of autism. Pharmaceuticals. (2022) 15:929. doi: 10.3390/ph15080929 36015079 PMC9414676

[248] Eissa N Awad MA Thomas SD Venkatachalam K Jayaprakash P Zhong S. Simultaneous antagonism at H3R/D2R/D3R reduces autism-like self-grooming and aggressive behaviors by mitigating MAPK activation in mice. Int J Mol Sci. (2022) 24:526. doi: 10.3390/ijms24010526 36613969 PMC9820264

[249] Cellot G Maggi L Di Castro MA Catalano M Migliore R Migliore M. Premature changes in neuronal excitability account for hippocampal network impairment and autistic-like behavior in neonatal BTBR T+tf/J mice. Sci Rep. (2016) 6:31696. doi: 10.1038/srep31696 27526668 PMC4985660

[250] Zhang L Xu X Ma L Wang X Jin M Li L. Zinc water prevents autism-like behaviors in the BTBR mice. Biol Trace Elem Res. (2023) 201:4779–92. doi: 10.1007/s12011-022-03548-1 PMC10415509 36602746

[251] Reichova A Bacova Z Bukatova S Kokavcova M Meliskova V Frimmel K. Abnormal neuronal morphology and altered synaptic proteins are restored by oxytocin in autism-related SHANK3 deficient model. Mol Cell Endocrinol. (2020) 518:110924. doi: 10.1016/j.mce.2020.110924 32619581

[252] Matsuo K Shinoda Y Abolhassani N Nakabeppu Y Fukunaga K. Transcriptome analysis in hippocampus of rats prenatally exposed to valproic acid and effects of intranasal treatment of oxytocin. Front Psychiatry. (2022) 13:859198. doi: 10.3389/fpsyt.2022.859198 35432011 PMC9005872

[253] Tian Y Yabuki Y Moriguchi S Fukunaga K Mao PJ Hong LJ. Melatonin reverses the decreases in hippocampal protein serine/threonine kinases observed in an animal model of autism. J Pineal Res. (2014) 56:1–11. doi: 10.1111/jpi.12081 23952810

[254] Liu Z Wang J Xu Q Wu Z You L Hong Q. Vitamin A supplementation ameliorates prenatal valproic acid-induced autism-like behaviors in rats. Neurotoxicology. (2022) 91:155–65. doi: 10.1016/j.neuro.2022.05.008 35594946

[255] Choi CH Schoenfeld BP Bell AJ Hinchey J Rosenfelt C Gertner MJ. Multiple drug treatments that increase cAMP signaling restore long-term memory and aberrant signaling in fragile X syndrome models. Front Behav Neurosci. (2016) 10:136. doi: 10.3389/fnbeh.2016.00136 27445731 PMC4928101

[256] Fuchs C Gennaccaro L Ren E Galvani G Trazzi S Medici G. Pharmacotherapy with sertraline rescues brain development and behavior in a mouse model of CDKL5 deficiency disorder. Neuropharmacology. (2020) 167:107746. doi: 10.1016/j.neuropharm.2019.107746 31469994

[257] Gioia R Seri T Diamanti T Fimmanò S Vitale M Ahlenius H. Adult hippocampal neurogenesis and social behavioural deficits in the R451C Neuroligin3 mouse model of autism are reverted by the antidepressant fluoxetine. J Neurochem. (2023) 165:318–33. doi: 10.1111/jnc.15753 36583243

[258] Zhou J Blundell J Ogawa S Kwon CH Zhang W Sinton C. Pharmacological inhibition of mTORC1 suppresses anatomical, cellular, and behavioral abnormalities in neural-specific Pten knock-out mice. J Neurosci. (2009) 29:1773–83. doi: 10.1523/JNEUROSCI.5685-08.2009 PMC3904448 19211884

[259] Tyzio R Nardou R Ferrari DC Tsintsadze T Shahrokhi A Eftekhari S. Oxytocin-mediated GABA inhibition during delivery attenuates autism pathogenesis in rodent offspring. Science. (2014) 343:675–9. doi: 10.1126/science.1247190 24503856

[260] Kim JW Seung H Kim KC Gonzales ELT Oh HA Yang SM. Agmatine rescues autistic behaviors in the valproic acid-induced animal model of autism. Neuropharmacology. (2017) 113:71–81. doi: 10.1016/j.neuropharm.2016.09.014 27638451

[261] Steinmetz AB Stern SA Kohtz AS Descalzi G Alberini CM. Insulin-like growth factor II targets the mTOR pathway to reverse autism-like phenotypes in mice. J Neurosci. (2018) 38:1015–29. doi: 10.1523/JNEUROSCI.2010-17.2017 PMC5783959 29217683

[262] Mottolese N Uguagliati B Tassinari M Cerchier CB Loi M Candini G. Voluntary running improves behavioral and structural abnormalities in a mouse model of CDKL5 deficiency disorder. Biomolecules. (2023) 13:1396. doi: 10.3390/biom13091396 37759796 PMC10527551

[263] Pinar C Yau SY Sharp Z Shamei A Fontaine CJ Meconi AL. Effects of voluntary exercise on cell proliferation and neurogenesis in the dentate gyrus of adult FMR1 knockout mice. Brain Plast. (2018) 4:185–95. doi: 10.3233/BPL-170052 PMC6311353 30598869

[264] Jamal I Kumar V Vatsa N Singh BK Shekhar S Sharma A. Environmental enrichment improves behavioral abnormalities in a mouse model of angelman syndrome. Mol Neurobiol. (2017) 54:5319–26. doi: 10.1007/s12035-016-0080-3 27581300

[265] Kentner AC Khoury A Lima Queiroz E MacRae M. Environmental enrichment rescues the effects of early life inflammation on markers of synaptic transmission and plasticity. Brain Behav Immun. (2016) 57:151–60. doi: 10.1016/j.bbi.2016.03.013 27002704

[266] Haratizadeh S Ranjbar M Basiri M Nozari M. Astrocyte responses to postnatal erythropoietin and nano-erythropoietin treatments in a valproic acid-induced animal model of autism. J Chem Neuroanat. (2023) 130:102257. doi: 10.1016/j.jchemneu.2023.102257 36918074

[267] Blokland A Heckman P Vanmierlo T Schreiber R Paes D Prickaerts J. Phosphodiesterase type 4 inhibition in CNS diseases. Trends Pharmacol Sci. (2019) 40:971–85. doi: 10.1016/j.tips.2019.10.006 31704172

[268] Choi CH Schoenfeld BP Weisz ED Bell AJ Chambers DB Hinchey J. PDE-4 inhibition rescues aberrant synaptic plasticity in Drosophila and mouse models of fragile X syndrome. J Neurosci. (2015) 35:396–408. doi: 10.1523/JNEUROSCI.1356-12.2015 25568131 PMC4287155

[269] Balakrishnan S Niebert M Richter DW. Rescue of cyclic AMP mediated long term potentiation impairment in the hippocampus of mecp2 knockout. (Mecp2(-/y) ) mice by rolipram. Front Cell Neurosci. (2016) 10:15. doi: 10.3389/fncel.2016.00015 26869885 PMC4737891

[270] Hagerman RJ Berry-Kravis E Kaufmann WE Ono MY Tartaglia N Lachiewicz A. Advances in the treatment of fragile X syndrome. Pediatrics. (2009) 123:378–90. doi: 10.1542/peds.2008-0317 PMC2888470 19117905

[271] Arzuaga AL Edmison DD Mroczek J Larson J Ragozzino ME. Prenatal stress and fluoxetine exposure in mice differentially affect repetitive behaviors and synaptic plasticity in adult male and female offspring. Behav Brain Res. (2023) 436:114114. doi: 10.1016/j.bbr.2022.114114 36116737

[272] Qiu W Go KA Wen Y Duarte-Guterman P Eid RS Galea LAM. Maternal fluoxetine reduces hippocampal inflammation and neurogenesis in adult offspring with sex-specific effects of periadolescent oxytocin. Brain Behav Immun. (2021) 97:394–409. doi: 10.1016/j.bbi.2021.06.012 34174336

[273] Winden KD Ebrahimi-Fakhari D Sahin M. Abnormal mTOR activation in autism. Annu Rev Neurosci. (2018) 41:1–23. doi: 10.1146/annurev-neuro-080317-061747 29490194

[274] Dai Y Zhang L Yu J Zhou X He H Ji Y. Improved symptoms following bumetanide treatment in children aged 3-6 years with autism spectrum disorder: a randomized, double-blind, placebo-controlled trial. Sci Bull. (2021) 66:1591–8. doi: 10.1016/j.scib.2021.01.008 36654288

[275] Cloarec R Riffault B Dufour A Rabiei H Gouty-Colomer LA Dumon C. Pyramidal neuron growth and increased hippocampal volume during labor and birth in autism. Sci Adv. (2019) 5:eaav0394. doi: 10.1126/sciadv.aav0394 30746473 PMC6357736

[276] Savardi A Borgogno M Narducci R La Sala G Ortega JA Summa M. Discovery of a small molecule drug candidate for selective NKCC1 inhibition in brain disorders. Chem. (2020) 6:2073–96. doi: 10.1016/j.chempr.2020.06.017 PMC7427514 32818158

[277] Doğan M Albayrak Y Erbaş O. Torasemide improves the propionic acid-induced autism in rats: A histopathological and imaging study. Alpha Psychiatry. (2023) 24:22–31. doi: 10.5152/alphapsychiatry.2023.22975 36879996 PMC9984905

[278] Purgert CA Izumi Y Jong YJ Kumar V Zorumski CF O'Malley KL. Intracellular mGluR5 can mediate synaptic plasticity in the hippocampus. J Neurosci. (2014) 34:4589–98. doi: 10.1523/JNEUROSCI.3451-13.2014 PMC3965784 24672004

[279] Arrang JM Garbarg M Schwartz JC. Auto-inhibition of brain histamine release mediated by a novel class (H3) of histamine receptor. Nature. (1983) 302:832–7. doi: 10.1038/302832a0 6188956

[280] Schaevitz LR Moriuchi JM Nag N Mellot TJ Berger-Sweeney J. Cognitive and social functions and growth factors in a mouse model of Rett syndrome. Physiol Behav. (2010) 100:255–63. doi: 10.1016/j.physbeh.2009.12.025 20045424

[281] Tropea D Giacometti E Wilson NR Beard C McCurry C Fu DD. Sur M. Partial reversal of Rett Syndrome-like symptoms in MeCP2 mutant mice. Proc Natl Acad Sci USA. (2009) 106:2029–34. doi: 10.1073/pnas.0812394106 PMC2644158 19208815

[282] Bozdagi O Tavassoli T Buxbaum JD. Insulin-like growth factor-1 rescues synaptic and motor deficits in a mouse model of autism and developmental delay. Mol Autism. (2013) 4:9. doi: 10.1186/2040-2392-4-9 23621888 PMC3649942

[283] Pizzarelli R Pimpinella D Jacobs C Tartacca A Kullolli U Monyer H. Insulin-like growth factor 2 (IGF-2) rescues social deficits in NLG3-/y mouse model of ASDs. Front Cell Neurosci. (2024) 17:1332179. doi: 10.3389/fncel.2023.1332179 38298376 PMC10827848

[284] Cruz E Descalzi G Steinmetz A Scharfman HE Katzman A Alberini CM. CIM6P/IGF-2 receptor ligands reverse deficits in angelman syndrome model mice. Autism Res. (2021) 14:29–45. doi: 10.1002/aur.2418 33108069 PMC8579913

[285] Ji C Yang J Lin L Chen S. Executive function improvement for children with autism spectrum disorder: A comparative study between virtual training and physical exercise methods. Children. (2022) 9:507. doi: 10.3390/children9040507 35455551 PMC9029765

[286] Alipour V Shabani R Rahmani-Nia F Vaseghi S Nasehi M Zarrindast MR. Effects of treadmill exercise on social behavior in rats exposed to thimerosal with respect to the hippocampal level of gluN1, gluN2A, and gluN2B. J Mol Neurosci. (2022) 72:1345–57. doi: 10.1007/s12031-022-02027-5 35597884

[287] Aronoff E Hillyer R Leon M. Environmental enrichment therapy for autism: outcomes with increased access. Neural Plast. (2016) 2016:2734915. doi: 10.1155/2016/2734915 27721995 PMC5046013

